# Charge Steering in Heterojunction Photocatalysis: General Principles, Design, Construction, and Challenges

**DOI:** 10.1002/smsc.202200041

**Published:** 2023-01-04

**Authors:** Mesfin Eshete, Xiyu Li, Li Yang, Xijun Wang, Jinxiao Zhang, Liyan Xie, Linjie Deng, Guozhen Zhang, Jun Jiang

**Affiliations:** ^1^ Hefei National Research Center for Physical Sciences at the Microscale School of Chemistry and Materials Science University of Science and Technology of China Jinzhai Road 96 Hefei Anhui 230026 P. R. China; ^2^ Department of Industrial Chemistry College of Applied Sciences Nanotechnology Excellence Center Addis Ababa Science and Technology University P.O. Box 16417 Addis Ababa Ethiopia; ^3^ College of Chemistry and Bioengineering Guilin University of Technology 12 Jian'gan Road Guilin Guangxi 541004 P. R. China; ^4^ A Key Laboratory of the- Ministry of Education for Advanced- Catalysis Materials Department of Chemistry Zhejiang Normal University Jinhua Zhejiang 321004 P. R. China

**Keywords:** graphene, heterojunctions, photocatalysis, Schottky/ohmic junctions, semiconductors

## Abstract

Steering charge kinetics is a key to optimizing quantum efficiency. Advancing the design of photocatalysts (ranging from single semiconductor to multicomponent semiconductor junctions) that promise improved photocatalytic performance for converting solar to chemical energy, entails mastery of increasingly more complicated processes. Indeed, charge kinetics become more complex as both charge generation and charge consumption may occur simultaneously on different components, generally with charges being transferred from one component to another. Capturing detailed charge dynamics information in each heterojunction would provide numerous significant benefits for applications and has been needed for a long time. Here, the steering of charge kinetics by modulating charge energy states in the design of semiconductor–metal‐interface‐based heterogeneous photocatalysts is focused. These phenomena can be delineated by separating heterojunctions into classes exhibiting either Schottky/ohmic or plasmonic effects. General principles for the design and construction of heterojunction photocatalysts, including recent advances in the interfacing of semiconductors with graphene, carbon quantum dots, and graphitic carbon nitride are presented. Their limitations and possible future outlook are brought forward to further instruct the field in designing highly efficient photocatalysts.

## Graphical abstract

Steering charge kinetics is a key to optimizing quantum efficiency. Advancing the design of photocatalysts that promise improved photocatalytic performance for converting solar to chemical energy, entails mastery of increasingly more complicated processes.

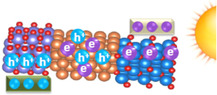

## 1 Introduction

Developing clean, low‐cost, and renewable fuel sources is a key challenge for meeting the energy demands of a fast‐growing population and increased industrialization, which at the same time presents a promising route for mitigating environmental issues caused by the combustion of fossil fuels.^[^ 1, 2, 3 ^]^ However, obtaining sufficient efficiency in large‐scale applications for converting solar energy into fuels and/or electricity is still a challenge. Holding so much promise for contributing to a sustainable energy future, photocatalytic splitting of water for the production of hydrogen and oxygen has received a great deal of attention from all over the world^[^ ^4^ ^]^ since the first demonstration by Fujishima and Honda using TiO_2_ and ultraviolet radiation (UV).^[^ ^5^ ^]^ As a result, several worthwhile single semiconductor photocatalysts utilizing Cu_2_O,^[^ 6, 7 ^]^ ZnO,^[^ ^8^ ^]^ WO_3_,^[^ 9, 10, 11 ^]^ etc. have been studied exhaustively aiming to optimize and achieve efficient semiconductor photocatalysis.

A semiconductor material is characterized by the highest occupied energy band (the valence band, VB), the lowest empty band (the conduction band, CB), and an energy bandgap (*E*_g_) separating the two, i.e., the energy region where no electronic states exist due to quantization of energy. Semiconductor‐based photocatalysis generally depends upon the generation of photoexcited charge carriers. Specifically, electrons at the VB maximum of the semiconductor are driven to the CB minimum by incident light, leaving oxidative holes at the VB maximum. In general, the process of semiconductor‐based photocatalysis consists of four main steps: 1) absorption of photons with energies equal to or greater than the bandgap; 2) charge separation of photoexcited electron–hole pairs in the bulk catalyst; 3) migration of charge carriers from the bulk to active sites on the surface of the catalyst; and 4) occurrence of chemical redox reactions (such as the splitting of water into hydrogen and oxygen) driven by energetic electrons or holes at active sites.^[^ 2, 4, 12, 13 ^]^

However, in the case of single semiconductors, there exist severe performance bottlenecks preventing their use in practical photocatalytic applications. For example, there are mutual tradeoffs between the energy region of the solar spectrum to which the semiconductor responds and the energetics of the reduction/oxidation (redox) reactions it is capable of catalyzing. Photons cannot generate e–h pairs unless their energies exceed or equal the bandgap (*E*_g_) of the semiconductor. It is also necessary to adjust band structures to enable a response to a wider region of the solar spectrum to achieve higher efficiency. The viability of the overall photocatalytic reaction requires the position of the CB (VB) edge to be higher (lower) than the potential of the reduction (oxidation) half‐reaction, respectively. For instance, in the splitting of water, the bottom of the CB should be at a more negative potential than the H^+^ to H_2_ reduction potential (0 V vs NHE at pH = 0), whereas the top of the VB must be located at a value more positive than the H_2_O to O_2_ oxidation potential (1.23 V vs NHE at pH = 0), making many otherwise promising semiconductors unsuitable for water‐splitting applications.^[^ 14, 15 ^]^

In single semiconductors, the relationship between the energy of absorbed photons and the redox potential of charge carriers is irreconcilable and incompatible. For example, for TiO_2_, only the UV region (≈5% of the solar spectrum) can be utilized. Moreover, the migration of photogenerated electrons and holes in single semiconductors often results in a high probability of unwanted charge recombination and low efficiency of charge migration to redox sites, along with an increased probability of encountering defects (trapping centers). In contrast to bulk single semiconductor photocatalysts, low‐dimensional crystals have charge carriers that can travel thousands of interatomic distances without scattering. The reduced dimensionality of these crystals maximizes not only their surface area but also the surface per quantity of electrons available for enhanced photocatalytic activity.^[^ 16, 17 ^]^ In general, low‐dimensional materials have shown appreciable differences in minimizing the recombination rate of electron–hole pairs as well as other electronic, catalytic, optical, and mechanical properties compared to their bulk counterparts.^[^ 18, 19, 20, 21, 22, 23, 24 ^]^

Overall, the key issues for enhancing photocatalytic performance are the improvement of the light absorption characteristics of catalytic systems, the enhancement of the separation of effective charge carriers, and the enlargement of catalytic surface area— all of which are limited by the intrinsic nature of single semiconductors. Additional critical factors required by practical applications include high chemical stability, high precision and flexibility of combinations of crystal structures and defects, optimum photocatalyst band positions, and low‐cost. To overcome the shortcomings of single semiconductors, creation of heterojunction structures has been proposed, such as by interfacing more than one semiconductor (photocatalyst), semiconductor–metal, semiconductor–semiconductor–metal, or semiconductor–metal–semiconductor layers in combination.

## 2 Charge Steering in Heterojunction Photocatalysis

The fundamental principle of building a semiconductor‐based photocatalytic heterostructure is to make full use of the advantages of each component by rationally arranging component geometry to maximize overall photocatalytic performance. Heterojunctions of p–n semiconductor (p–n), semiconductor–metal (s–m), semiconductor–semiconductor–metal, and Z‐scheme semiconductor (Z‐scheme) hybrid systems are the basic structures of well‐documented designs for effective photocatalytic systems. The improved photocatalytic efficiency of these is mainly attributed to increased rates of charge separation and migration and utilization of a greater portion of the broad solar spectrum. Establishing relationships between the parameters characterizing these heterojunctions within each reaction step is important for gaining charge kinetics information of a given photocatalytic system.

To improve the design of heterojunction photocatalysts, tuning charge kinetics dynamics to improve charge separation and minimize loss of energy during charge carrier migration is critical for quantum yield optimization. Designs of multicomponent semiconductor junctions have long sought to steer charge flows and attain more efficient charge separation. Inherently, the charge kinetics becomes more complicated in these systems because both charge generation, as well as charge consumption, may simultaneously take place on different components and because charge is generally transferred from one component to another. However, capturing detailed charge dynamics information at each heterojunction benefits numerous important applications.

This review focuses on the steering of charge kinetics in different semiconductor heterojunction systems to improve charge separation as determined by the nature of the generated internal electric field and characteristics of band bending at the junction. Further, it covers recent work on heterojunctions including: 1) Schottky/ohmic junctions and plasmonic effects models; 2) materials incorporating semiconductor–graphene, semiconductor(S)–graphitic carbon nitride (C_3_N_4_), semiconductor–(RGO/metal)–graphitic carbon nitride (g–C_3_N_4_); 3) recent fascinating investigations of CQDs incorporated into graphitic carbon nitride heterojunctions, and the advances achieved by each for enhancing the overall photocatalytic process.

## 3 p‐Type–n‐Type (p–n) Junction photocatalysis

**Figure 1:**
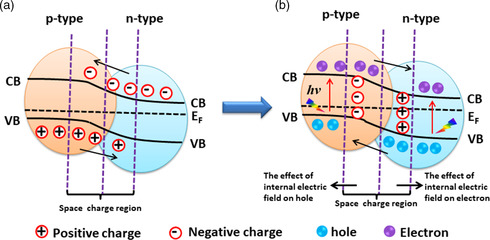
a,b) Schematic representations of a p–n heterojunction between two semiconductors without an externally applied voltage (a), and electron–hole separation in a p–n heterojunction photocatalyst under the influence of the internal electric field upon light illumination (b).

In the design of multicomponent semiconductor junctions, the steering of charge flow has long been recognized as the key for creating efficient charge separation. Yongsheng et al. first showed that the p–n heterojunction enhances photocatalytic activity more efficiently than conventional heterojunctions (i.e., heterojunctions composed of different materials or the same materials (see **Figure** **1**) n‐type–n‐type or p‐type–p‐type heterojunctions);^[^ ^25^ ^]^ subsequently, this finding has been confirmed by many others.^[^ 2, 15, 26, 27, 28 ^]^

The basic principle of a p–n system consists of two different components—a p‐type and an n‐type semiconductor—that are in direct contact, as depicted in Figure 1a. Each electron from the n‐type semiconductor that diffuses into the p‐type semiconductor leaves a positive charge behind; similarly, a hole migrating from the p‐type to the n‐type semiconductor leaves a negative charge. Electron–hole diffusion continues until the system achieves Fermi‐level equilibrium. As a result, a charged region forms close to the p–n interface, the so‐called internal electric field. With the formation of the internal electric field, after light excitation, photoexcited electrons transfer from high CB to low CB and holes from low VB to high VB, and these e–h pairs remain well separated, as shown in Figure 1b.

**Figure 2:**
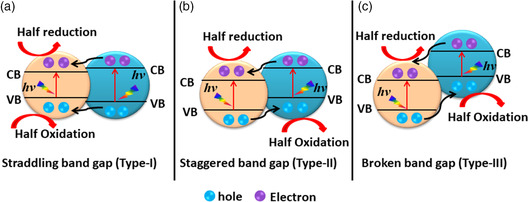
a–c) Schematic of the three different types of p–n junction photocatalysts: (a) Type I, (b) Type II, and (c) Type III upon light exposure.

Conventionally, semiconductor 1–semiconductor 2 hybrid junctions are classified as Type I, Type II, or Type III, as shown in **Figure** **2**. When a Type‐I p–n heterojunction is irradiated with light with energy greater than or equal to the bandgap, the photogenerated electrons migrate from the higher CB to the lower CB and the holes from lower VB to higher VB, as depicted in Figure 2a. Similarly, migration of photoexcited electrons and holes in Type‐II and Type‐III p–n junctions occurs from high CB to low CB and low VB to high VB, and effective charge separation can be obtained in either, as shown in Figure 2b,c. In the latter two types, separation results from the migration of high‐energy electrons and the opposite movement of high energy holes, whereas in the straddling bandgap (Type I), both high‐energy electrons and holes move to the same semiconductor, which disfavors improvement of photocatalytic activity.

Notably, Type‐II p–n heterojunction photocatalysts offer favorable band alignments for efficient charge carrier separation. Moreover, rapid charge transfer assisted by the internal electric field formed at the interface junction is beneficial for producing rates of charge separation that lead to enhanced photocatalytic reactions. One of the most investigated Type‐II p–n multicomponent junctions is the Cu_2_O/TiO_2_ heterojunction photocatalyst in which p‐type Cu_2_O and n‐type TiO_2_ contact directly.^[^ ^29^ ^]^ Recently, C. Ding et al. synthesized a highly efficient Cu_2_O–TiO_2_ heterojunction by a wet chemical method. The Cu_2_O–TiO_2_ heterojunction prepared in this way achieves an improved methylene blue degradation rate of 93.67% in 45 min, more efficient than pristine Cu_2_O. At the p–n junction, free electrons diffuse from TiO_2_ to Cu_2_O and holes from Cu_2_O to TiO_2_ until equilibrium is reached. Consequently, they leave negative and positive regions at the Cu_2_O/TiO_2_ interface, which forms a space charge region that results in an internal electric field. Upon irradiation of light, photoexcited electrons transfer from the CB of Cu_2_O (*E*_g_ = 2−2.2 eV)^[^ 30, 31, 32 ^]^ to the CB of TiO_2_ (*E*_g_ = 3.2 eV) and photoexcited holes migrate from the VB of TiO_2_ to the VB of Cu_2_O, a condition that favors reduction at TiO_2_ and oxidation at Cu_2_O. The formation of the internal electric field minimizes the recombination probability of photoexcited electron–hole pairs and results in improved photocatalytic activity, as illustrated in Figure 1b.

The study of p–n heterojunctions has been extended to 2D p–n junction nanosheets. The combined experimental and theoretical work of Nan et al. on MoS_2_/TiO_2_ (a Type‐II p–n junction), demonstrates that enhanced visible light absorption can result in enhanced photocatalytic activity.^[^ ^33^ ^]^ Their photocurrent density analysis shows that the MoS_2_/TiO_2_ heterojunction has 17.8 times higher activity than that of pristine TiO_2_. The photocatalytic degradation enhancement factor of the corresponding kinetic constant is about 5.2. Such a dramatic improvement originates: 1) from the incorporation of MoS_2_ nanosheets that improve light‐harvesting performance; and 2) from the favorable band alignment that results in fast and efficient charge separation of photogenerated charge carriers. Additional comprehensive studies of many outstanding p–n junction designs are outlined in **Table** **1**.

**Table 1:** Summary of representative p–n heterojunctions reported so far

Sample/model	Heterojunction class	Oxidation site	Reduction site	Charge migration direction across the interface	Activity test/Application	References
NiO–ZnO	Type II	NiO	ZnO	NiO 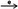 ZnO, ZnO 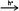 NiO	Degradation of rhodamine B (RhB)	[88, 89, 90, 91, 92, 93]
NiO–TiO_2_	Type II	NiO	TiO_2_	NiO 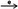 TiO_2_, TiO_2_ 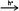 NiO	Photocatalytic reduction of Cr_2_O_7_^2−^ and photocatalytic oxidation of RhB,hydrogen generation	[94, 95, 96]
NiO–SnO_2_	Type II	NiO	SnO_2_	NiO 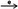 SnO_2_, SnO_2_ 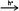 NiO	Degradation of RhB	[97]
Cu_2_O–TiO_2_	Type II	Cu_2_O	TiO_2_	Cu_2_O 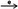 TiO_2_, TiO_2_ 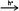 Cu_2_O	Decomposition of p‐Nitrophenol, Orange II oxidation, MB degradation, 2,4,6‐trichloro‐phenol degradation, enhanced photocurrents,hydrogen generation	[29, 98, 99, 100, 101, 102, 103, 104, 105, 106, 107, 108]
CuO–In_2_O_3_	Type II	CuO	In_2_O_3_	CuO 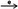 In_2_O_3_, In_2_O_3_ 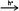 CuO	Degradation of RhB	[109]
ZnS–Cu_2_S	Type I	–	Cu_2_S	ZnS 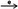 Cu_2_S	Photocatalytic water splitting	[110]
Cu_2_O–CdS	Type II	Cu_2_O	CdS	Cu_2_O 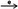 CdS, CdS 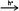 Cu_2_O	Hydrogen Evolution	[111, 112]
CuO–ZnO	Type II	CuO	ZnO	CuO 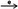 ZnO, ZnO 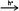 CuO	Photodegradation of Phenol, azo dye, (MB), degradation of Congo red, and benzoic acid, photocurrent production	[93, 113, 114, 115, 116, 117, 118]
CuO–SnO_2_	Type II	CuO	SnO_2_	CuO 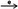 SnO_2_, SnO_2_ 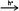 CuO	Decomposition of MB	[119]
Cu_2_O–ZnO	Type II	Cu_2_O	ZnO	Cu_2_O 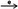 ZnO, ZnO 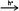 Cu_2_O	Methyl orange (MO) photodegradation, reduction of methylviologen (MV^2+^)	[120, 121, 122, 123, 124]
CuS–ZnO	Type II	CuS	ZnO	CuS 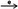 ZnO, ZnO 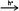 CuS	Decomposition of MB	[125]
CuS–TiO_2_	Type II	CuS	TiO_2_	CuS 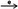 TiO_2_, TiO_2_ 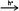 CuS	H_2_ production	[126]
CuS–CdS	Type II	CdS	CuS	CdS 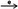 CuS, CuS 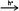 CdS	Degradation of MB and H_2_ production	[127, 128]
CuS–CdS	Type II	CuS	CdS	CuS 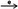 CdS, CdS 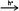 CuS	H_2_ production	[129]
Cu_2_S–CdS	Type II	Cu_2_S	CdS	Cu_2_S 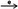 CdS, CdS 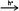 Cu_2_S	H_2_ production	[130]
Cu_2_S–ZnO	Type II	Cu_2_S	ZnO	Cu_2_S 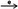 ZnO, ZnO 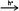 Cu_2_S	Hydrogen generation	[131]
Cu_3_P–CdS	Type II	Cu_3_P	CdS	Cu_3_P 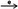 CdS, CdS 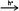 Cu_3_P	H_2_ production	[132]
Cu_3_P–TiO_2_	Type II	Cu_3_P	TiO_2_	Cu_3_P 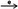 TiO_2,_ TiO_2_ 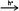 Cu_3_P	H_2_ production	[133]
Ag_2_O–TiO_2_	Type II	Ag_2_O	TiO_2_	Ag_2_O 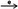 TiO_2,_ TiO_2_ 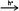 Ag_2_O	Degradation of MO	[134]
Ag_2_O–ZnO	Type II	Ag_2_O	ZnO	Ag_2_O 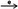 ZnO, ZnO 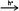 Ag_2_O	MB degradation	[135]
SnO–SnO_2_	Type II	SnO	SnO_2_	SnO 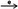 SnO_2,_ SnO_2_ 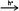 SnO	MB degradation	[136]
PbS –SnO_2_	Type II	PbS	SnO_2_	PbS 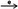 SnO_2,_ SnO_2_ 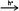 PbS	Degradation of RhB	[137]
SnO–ZnO	Type II	SnO	ZnO	SnO 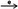 ZnO, ZnO 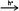 SnO	MB degradation	[138]
SnS–ZnO	Type II	SnS	ZnO	SnS 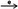 ZnO, ZnO 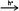 SnS	Degradation of MO, RhB	[139]
Ag_2_O–Bi_2_O_2_CO_3_	Type II	Ag_2_O	Bi_2_O_2_CO_3_	Ag_2_O 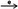 Bi_2_O_2_CO_3,_ Bi_2_O_2_CO_3_ 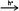 Ag_2_O	Degradation of RhB, MB, and MO	[140]
Ag_2_O–Bi_2_WO_6_	Type II	Ag_2_O	Bi_2_WO_6_	Ag_2_O 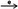 Bi_2_WO_6,_ Bi_2_WO_6_ 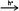 Ag_2_O	Degradation RhB	[141]
Co_3_O_4_/BiVO_4_	Type I	Co_3_O_4_	Co_3_O_4_	BiVO_4_ 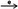 Co_3_O_4,_ BiVO_4_ 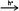 Co_3_O_4_	Degradation of Phenol,RhB	[142, 143]
*m*‐BiVO_4_‐γ‐Bi_2_O_3_	Type II	γ‐Bi_2_O_3_	*m*‐BiVO_4_	γ‐Bi_2_O_3_ 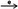 m‐BiVO_4,_ *m*‐BiVO_4_ 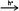 γ‐Bi_2_O_3_	Degradation RhB	[144, 145]
Co_3_O_4_/Fe_2_O_3_	Type II	Co_3_O_4_	Fe_2_O_3_	Co_3_O_4_ 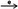 Fe_2_O_3,_ Fe_2_O_3_ 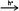 Co_3_O_4_	Water Splitting	[146]
Co_3_O_4_/WO_3_	Type II	Co_3_O_4_	WO_3_	Co_3_O_4_ 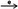 WO_3,_ WO_3_ 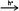 Co_3_O_4_	2‐propanol decomposition	[147]
CoP_3_/Ni_2_P	Type II	CoP_3_	Ni_2_P	CoP_3_ 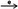 Ni_2_P, Ni_2_P 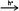 CoP_3_	H_2_ production	[148]
Bi_2_O_3_–TiO_2_	Type II	Bi_2_O_3_	TiO_2_	Bi_2_O_3_ 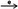 TiO_2,_ TiO_2_ 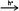 Bi_2_O_3_	Degradation of *p*‐chlorophenol, MB, pentachlorophenol	[99, 149]
Bi_2_O_3_–SnO_2_	Type II	Bi_2_O_3_‐	SnO_2_	Bi_2_O_3_ 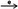 SnO_2,_ SnO_2_ 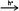 Bi_2_O_3_	Degradation of RhB	[150]
Bi_2_O_3_–ZnO	Type II	Bi_2_O_3_	ZnO	Bi_2_O_3_ 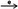 ZnO, ZnO 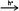 Bi_2_O_3_	Degradation of alizarin red (AR) dye	[151]
V_2_O_5_–TiO_2_	Type I/II	V_2_O_5_	TiO_2_	V_2_O_5_ 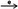 TiO_2,_ TiO_2_ 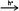 V_2_O_5_	RhB degradation, H_2_ production	[152, 153, 154]
CuO–TiO_2_	Type II	CuO	TiO_2_	CuO 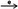 TiO_2,_ TiO_2_ 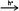 CuO	H_2_ production, Orange II oxidation	[155, 156, 157]
CeO_2_–TiO_2_	Type II	CeO_2_	TiO_2_	CeO_2_ 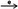 TiO_2,_ TiO_2_ 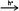 CeO_2_	Oxidative degradation of CV dye (Crystal Violet)	[158]
CeO_2_–Ag_3_PO_4_	Type II	CeO_2_	Ag_3_PO_4_	CeO_2_ 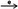 Ag_3_PO_4,_ Ag_3_PO_4_ 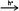 CeO_2_	Degradation of MB, RhB, and ciproﬂoxacin (CIP)	[159]
NiO–SrTiO_3_	Type II	NiO	SrTiO_3_	NiO 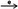 SrTiO_3,_ SrTiO_3_ 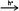 NiO	Enhanced photocurrents	[160]
CuO–BaTiO_3_	Type I	CuO	CuO	BaTiO_3_ 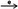 CuO, BaTiO_3_ 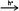 CuO	MO degradation	[161]
Cu_2_O–SrTiO_3_	Type II	Cu_2_O	SrTiO_3_	Cu_2_O 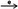 SrTiO_3,_ SrTiO_3_ 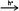 Cu_2_O	Photodegradation of tetracycline (TC)	[162]
CuO–PbTiO_3_	Type II	CuO	PbTiO_3_	CuO 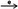 PbTiO_3,_ PbTiO_3_ 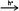 CuO	Degradation of malachite green	[163]
α‐Fe_2_O_3_/Cu_2_O	Type II	α‐Fe_2_O_3_	Cu_2_O	α‐Fe_2_O_3_ 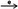 Cu_2_O, Cu_2_O 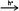 α‐Fe_2_O_3_	Photoreduction of CO_2_	[38]
α‐Fe_2_O_3_/ZnO	Type II	α‐Fe_2_O_3_	ZnO	α‐Fe_2_O_3_ 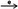 ZnO, ZnO 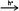 α‐Fe_2_O_3_	H_2_ production	[164]
α‐Fe_2_O_3_/CuPc	Type II	α‐Fe_2_O_3_	CuPc	α‐Fe_2_O_3_ 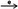 CuPc, CuPc 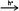 α‐Fe_2_O_3_	Photoreduction of CO_2_	[165]
Cu_2_O–Fe_2_O_3_	Type II	Cu_2_O	Fe_2_O_3_	Cu_2_O 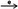 Fe_2_O_3,_ Fe_2_O_3_ 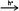 Cu_2_O	Degradation of MB and RhB	[166]
MoS_2_–CeO_2_	Type II	MoS_2_	CeO_2_	MoS_2_ 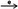 CeO_2,_ CeO_2_ 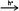 MoS_2_	H_2_ production	[167]
MoS_2_–CdS	Type I	MoS_2_	MoS_2_	CdS 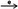 MoS_2,_ CdS 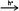 MoS_2_	H_2_ production	[168, 169, 170]
MoS_2_–TiO_2_	Type II	MoS_2_	TiO_2_	MoS_2_ 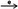 TiO_2,_ TiO_2_ 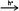 MoS_2_	Photodegradation of 4‐CP, RhB, hydrogen evolution	[33, 127, 171]
MoS_2_–TiO_2_	Type II	TiO_2_	MoS_2_	TiO_2_ 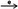 MoS_2,_ MoS_2_ 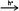 TiO_2_	Hydrogen evolution	[172]
MoS_2_–ZrO_2_	Type I	MoS_2_	MoS_2_	ZrO_2_ 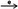 MoS_2,_ ZrO_2_ 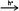 MoS_2_	Degradation of MO	[173]
MoS_2_–SnO_2_	Type II	MoS_2_	SnO_2_	MoS_2_ 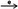 SnO_2,_ SnO_2_ 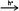 MoS_2_	Methylene blue (MB) degradation	[174]
MoS_2_–WO_3_	Type II	MoS_2_	WO_3_	MoS_2_ 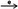 WO_3,_ WO_3_ 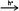 MoS_2_	Degradation of Congo red (CR)	[175]
MoS_2_–ZnS	Type I	MoS_2_	MoS_2_	ZnS 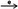 MoS_2,_ ZnS 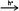 MoS_2_	Malachite green dye degradation	[176]
α‐MoO_3_–MoS_2_	Type II	MoS_2_	α‐MoO_3_	MoS_2_ 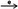 α‐MoO_3,_ α‐MoO_3_ 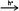 MoS_2_	RhB degradation	[177]
MoS_2_–WSe_2_	Type II	MoS_2_	WSe_2_	MoS_2_ 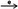 WSe_2,_ WSe_2_ 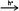 MoS_2_	Hydrogen evolution	[178, 179]
BiOI–TiO_2_	Type I	BiOI	BiOI	TiO_2_ 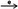 BiOI, TiO_2_ 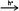 BiOI	MO degradation	[180, 181]
BiOBr–TiO_2_	Type II	TiO_2_	BiOBr	TiO_2_ 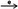 BiOBr, BiOBr 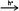 TiO_2_	Degradation RhB and MO	[182, 183]
BiOI–SnS_2_	Type II	BiOI	SnS_2_	BiOI 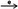 SnS_2,_ SnS_2_ 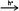 BiOI	RhB degradation	[184]
BiOI–TiO_2_	Type II	BiOI	TiO_2_	BiOI 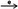 TiO_2,_ TiO_2_ 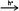 BiOI	MO degradation, RhB degradation	[185, 186]
BiOI–WO_3_	Type II	BiOI	WO_3_	BiOI 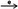 WO_3,_ WO_3_ 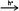 BiOI	Degradation tetracycline (TC)	[187]
ZnMn_2_O_4_–TiO_2_	Type I	ZnMn_2_O_4_	ZnMn_2_O_4_	TiO_2_ 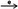 ZnMn_2_O_4,_ TiO_2_ 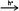 ZnMn_2_O_4_	Orange II oxidation	[99]
Ag_3_PO_4_–TiO_2_	Type II	Ag_3_PO_4_	TiO_2_	Ag_3_PO_4_ 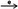 TiO_2_ TiO_2_ 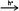 Ag_3_PO_4_	Degradation RhB and MB	[188]
Ag_3_PO_4_–BiVO_4_	Type II	Ag_3_PO_4_	BiVO_4_	Ag_3_PO_4_ 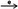 BiVO_4,_ BiVO_4_ 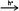 Ag _3_PO_4_	Degradation RhB and MB	[189]
CuInS_2_–TiO_2_	Type II	CuInS_2_	TiO_2_	CuInS_2_ 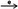 TiO_2,_ TiO_2_ 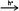 CuInS_2_	4‐nitrophenol degradation	[190]
Bi_12_TiO_20_–TiO_2_	Type II	Bi_12_TiO_20_	TiO_2_	Bi_12_TiO_20_ 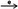 TiO_2,_ TiO_2_ 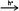 Bi_12_TiO_20_	RhB degradation	[191]
YFeO_3_–TiO_2_	Type II	YFeO_3_	TiO_2_	YFeO_3_ 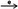 TiO_2,_ TiO_2_ 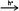 YFeO_3_	Benzene oxidation, Orange II oxidation	[192]
CaFe_2_O_4_/MgFe_2_O_4_	Type II	CaFe_2_O_4_	MgFe_2_O_4_	CaFe_2_O_4_ 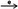 MgFe_2_O_4,_ MgFe_2_O_4_ 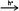 CaFe_2_O_4_	Degradation of isopropyl alcohol hydrogen evolution	[193]
BiOCl–SrFe_12_O_19_	Type I	SrFe_12_O_19_	SrFe_12_O_19_	BiOCl 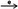 SrFe_12_O_19,_ 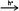 BiOClSrFe_12_O_19_	MB degradation	[194]
BiOCl–TiO_2_	Type II	TiO_2_	BiOCl	TiO_2_ 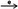 BiOCl, BiOCl 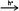 TiO_2_	MB degradation	[195]
BiOCl–SnO_2_	Type II	BiOCl	SnO_2_	BiOCl 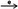 SnO_2,_ SnO_2_ 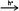 BiOCl	RhB degradation	[196]
BiOCl–BiVO_4_	Type II	BiOCl	BiVO_4_	BiOCl 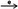 BiVO_4,_ BiVO_4_ 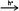 BiOCl	MO photodegradation	[197]
BiOI–CdWO_4_	Type II	BiOI	CdWO_4_	BiOI 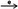 CdWO_4,_ CdWO_4_ 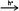 BiOI	Degradation of TC and RhB	[198]
BiOBr–ZnO	Type II	BiOBr	ZnO	BiOBr 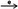 ZnO, ZnO 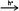 BiOBr	MO decolorization	[199]
BiOI–ZnO	Type II	BiOI	ZnO	BiOI 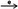 ZnO, ZnO 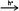 BiOI	Degradation of CR,MO	[200, 201]
BiOBr–CeO_2_	Type II	CeO_2_	BiOBr	CeO_2_ 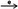 BiOBr, BiOBr 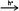 CeO_2_	Degradation of RhB, MB, and bisphenol A (BPA)	[202]
BiOBr–WO_3_	Type II	BiOBr	WO_3_	BiOBr 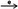 WO_3,_ WO_3_ 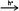 BiOBr	Degradation of RhB, MO, and *para*‐chlorophenol (4‐CP)	[203]
BiOBr–BiVO_4_	Type II	BiOBr	BiVO_4_	BiOBr 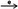 BiVO_4,_ BiVO_4_ 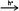 BiOBr	Degradation of MB	[204, 205]
BiOBr–BiVO_4_	Type I	BiVO_4_	BiVO_4_	BiOBr 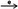 BiVO_4,_ BiOBr 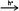 BiVO_4_	Degradation of MB	[206]
Ag_3_VO_4_–WO_3_	Type II	Ag_3_VO_4_	WO_3_	Ag_3_VO_4_ 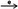 WO_3,_ WO_3_ 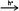 Ag_3_VO_4_	Degradation of TC	[159]
Bi_2_O_3_–WO_3_	Type II	Bi_2_O_3_	WO_3_	Bi_2_O_3_ 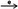 WO_3,_ WO_3_ 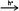 Bi_2_O_3_	Decomposition of RhB and 4‐nitroaniline (4‐NA)	[207]
Bi_2_O_3_–BaTiO_3_	Type II	BaTiO_3_	Bi_2_O_3_	BaTiO_3_ 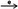 Bi_2_O_3,_ Bi_2_O_3_ 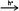 BaTiO_3_	Degradations of MO and MB	[208]
AgBr–BiPO_4_	Type II	AgBr	BiPO_4_	AgBr 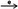 BiPO_4,_ BiPO_4_ 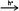 AgBr	MB degradation	[209]
Ag_3_PO_4_–AgBr	Type II	AgBr	Ag_3_PO_4_	AgBr 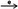 Ag_3_PO_4,_ Ag_3_PO_4_ 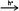 AgBr	Degradation of MB and RhB	[210, 211]
BiOI–La_2_Ti_2_O_7_	Type II	BiOI	La_2_Ti_2_O_7_	BiOILa_2_ 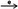 Ti_2_O_7,_ La_2_Ti_2_O_7_ 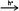 BiOI	Degradation of RhB, dye X‐3B, MO	[212]
Cu_2_O–Ta_2_O_5_	Type II	Cu_2_O	Ta_2_O_5_	Cu_2_O 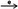 Ta_2_O_5,_ Ta_2_O_5_ 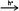 Cu_2_O	Generated hydrogen and oxygen	[213]
CuCrO_2_–SnO_2_	Type II	CuCrO_2_	SnO_2_	CuCrO_2_ 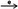 SnO_2,_ SnO_2_ 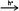 CuCrO_2_	Degradation of MB	[214]
CuCo_2_O_4_–TiO_2_	Type II	CuCo_2_O_4_	TiO_2_	CuCo_2_O_4_ 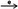 TiO_2,_ TiO_2_ 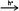 CuCo_2_O_4_	Hydrogen evolution, photocurrent density	[215]
CdS–LaFeO_3_	Type II	CdS	LaFeO_3_	CdS 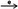 LaFeO_3,_ LaFeO_3_ 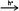 CdS	Degradation MB, RhB, and MO	[216]
CuO–BiFeO_3_	Type II	CuO	BiFeO_3_	CuO 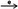 BiFeO_3,_ BiFeO_3_ 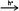 CuO	Degradation of MO	[217]
BiFeO_3_–ZnO	Type II	BiFeO_3_	ZnO	BiFeO_3_ 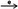 ZnO, ZnO 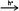 BiFeO_3_	Photodegradation of 2,4‐dichlorophenol and RhB	[218]
BiVO_4_–Mn_3_O_4_	Type II	Mn_3_O_4_	BiVO_4_	Mn_3_O_4_ 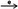 BiVO_4,_ BiVO_4_ 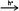 Mn_3_O_4_	Degradation MB	[219]
BiOI–ZnTiO_3_	Type II	BiOI	ZnTiO_3_	BiOI 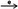 ZnTiO_3,_ ZnTiO_3_ 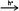 BiOI	Degradation of Rhodamine 6 G	[220]
LaCrO_3_–PbTiO_3_	Type II	LaCrO_3_	PbTiO_3_	LaCrO_3_ 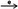 PbTiO_3,_ PbTiO_3_ 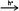 LaCrO_3_	Degradation of phenol	[221]
CdS–Ni_2_P	Type II	CdS	Ni_2_P	CdS 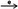 Ni_2_P, Ni_2_P 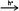 CdS	Hydrogen Evolution	[222]
BiVO_4_@Cu_3_SnS_4_	Type II	BiVO_4_	Cu_3_SnS_4_	BiVO_4_ 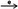 Cu_3_SnS_4,_ Cu_3_SnS_4_ 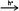 BiVO_4_	Degradation MB	[223]

Even though better charge separation and improved photocatalysis are attained, some disadvantages do limit the photocatalytic performance of p–n heterojunctions. The joining of p‐type and n‐type semiconductors to build p–n junctions is negatively impacted by crystal lattice mismatching, which results in poor coupling at the interface and quenching of charge carrier migration. At the same time, migration of the energetic carriers that guarantee effective charge separation is achieved at the cost of partial loss of the energy obtained from the photons absorbed. This energy consumption loss reduces the reduction and oxidation potential of charge carriers, thus lowering the photocatalytic reaction rate.

## 4 Semiconductor–Metal (s–m) Heterojunctions

**Figure 3:**
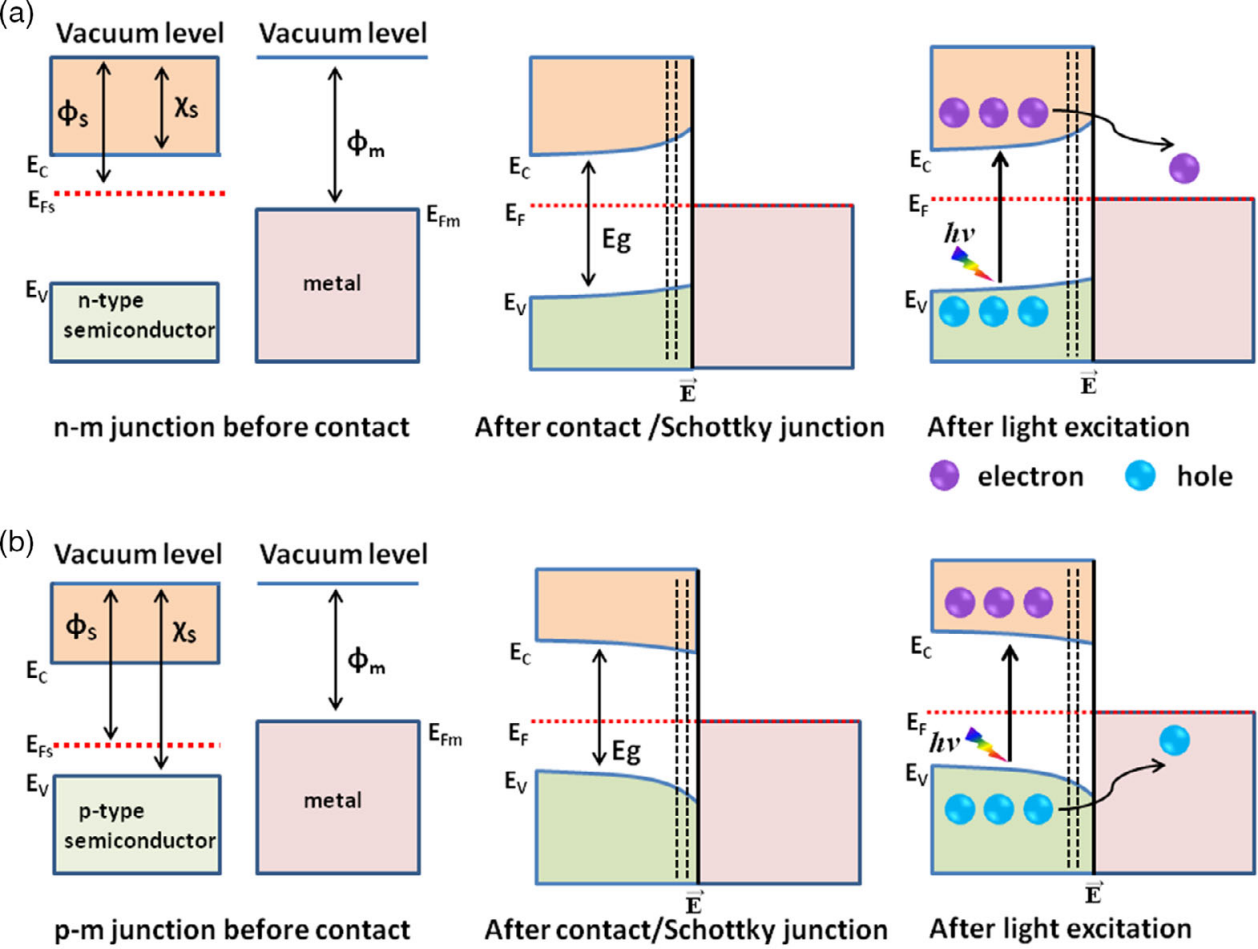
a) Schematic of n‐type‐metal Schottky junction formation, followed by light absorption and charge separation. b) Schematic of p‐type‐metal Schottky junction formation, followed by light absorption and charge separation.

Interfacing a semiconductor with metal is a well‐known design strategy aimed at improving charge separation. The integration of metals with semiconductors results in Schottky/ohmic or plasmon junctions depending on the relative work functions. The minimum energy required to remove an electron from the Fermi (*E*_f_) level to the vacuum level is defined as the work function. When n‐type/p‐type semiconductors with higher/lower work functions contact a metal directly, free electrons flow from the higher Fermi level to the lower Fermi level until the two Fermi levels equilibrate. The two main mechanisms involved at the interface of the s–m junction photocatalytic system can produce either a Schottky/ohmic junction or one characterized by plasmonic effects. The former can be formed whenever the work function difference between semiconductor and metal at the interface is appropriate. When an n‐type semiconductor has a lower work function than the metal contacting it (*ϕ*_m_ > *ϕ*_sn_), free electrons migrate from the lower work function (higher Fermi level) to the higher work function (lower Fermi level) until the Fermi levels equilibrate, resulting in an accumulation of negative charge—with positive charge remaining in the semiconductor layer due to electrostatic induction, as shown in **Figure** **3**a.

The electric ﬁeld formed at the metal–semiconductor interface cannot be screened eﬀectively in the semiconductor due to the low concentration of free charge carriers there. This causes the free charge carrier concentration near the semiconductor surface to be depleted compared with the bulk; a space charge region (also called the depletion layer) is formed on one side of the semiconductor, with the electric field direction pointing from semiconductor to metal. This makes the energy band bend upward, going from semiconductor to metal, thus forming the Schottky barrier. It should be noted that electron flow hardly affects *E*_fm_ due to the high density pool of free electrons in the metal. Instead, only the band level of the semiconductor shifts up or down. In the case of integrating, a p‐type semiconductor having a higher work function than the metal (*ϕ*_m_ < *ϕ*_sp_), as shown in Figure 3b, free electrons migrate from metal‐to‐semiconductor; positive charge accumulates in the metal and negative charge in the semiconductor, forming a space charge region that results in bending the band downward, and in an electric field that points from metal to semiconductor. Generally, a Schottky barrier may promote charge separation that results in the enhancement of photocatalytic performance by preventing the recombination of electron–hole pairs. Normally, most noble metals possess higher work functions than n‐type semiconductors and lower than p‐type semiconductors, thus favoring the formation of Schottky barriers.

**Figure 4:**
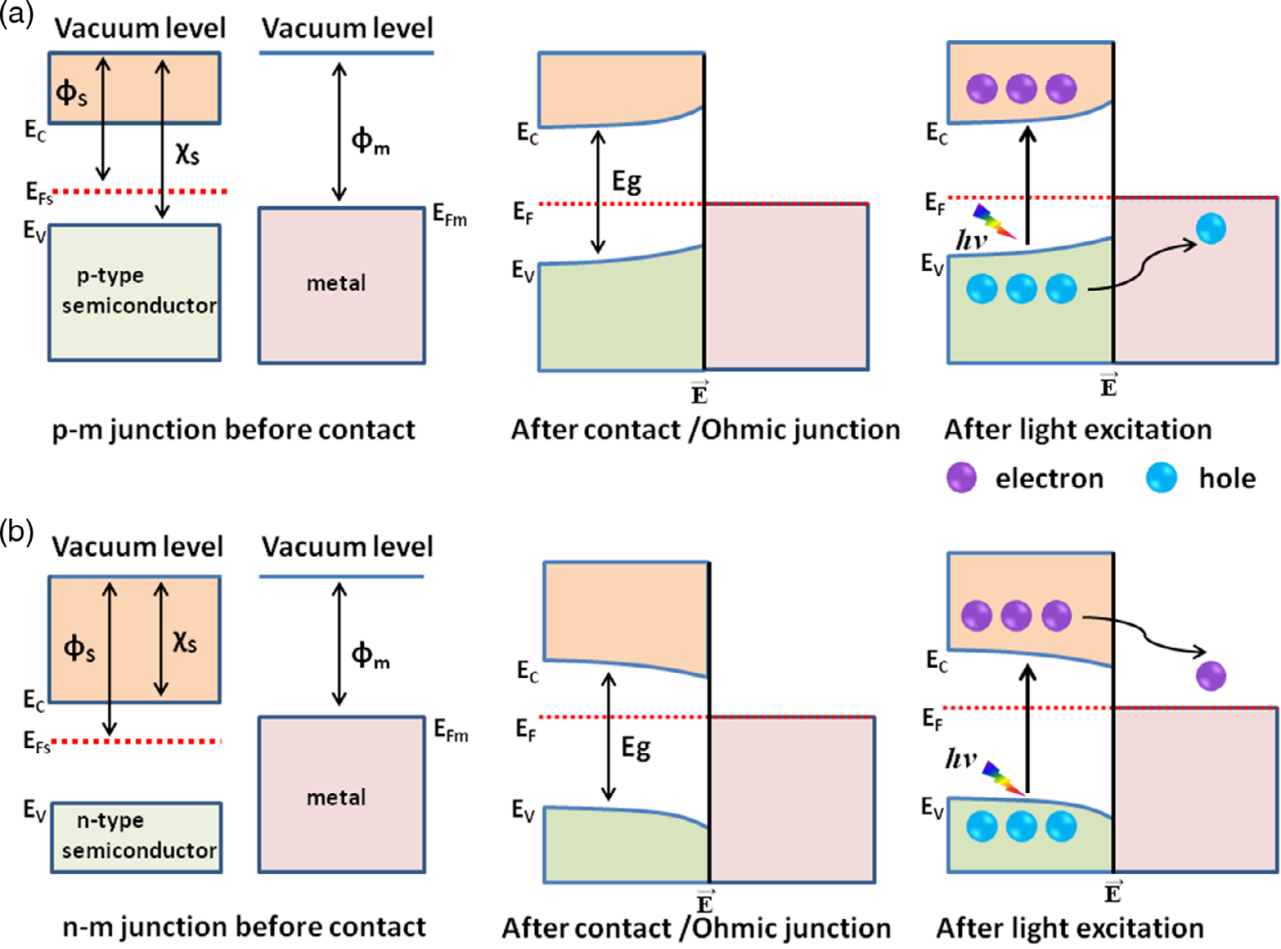
a) Schematic of p‐type‐metal ohmic junction formation, light absorption, and charge separation. b) Schematic of n‐type‐metal ohmic junction formation, light absorption, and charge separation.

In contrast, when metal is in contact with an n‐type semiconductor of higher work function or a p‐type semiconductor of lower work function (*ϕ*_m_ < *ϕ*_sn_ or *ϕ*_m_ > *ϕ*_sp_) free electrons from the n‐type (holes from the p‐type) semiconductor accumulate in the space charge region. The bands bend opposite the band in a Schottky junction, where no barrier is created between metal and semiconductor. The metal–semiconductor interface in the absence of a barrier results in an ohmic junction, as shown in **Figure** **4**. Generally, band bending and the creation of an inner electric field at the interface results in the migration and separation of low energy electrons (holes) to the metal and confines high energy charges to the semiconductors.^[^ 12, 34, 35 ^]^

Upon light absorption, photoexcited electrons from the CB of the n‐type semiconductor are transferred to the metal; this process induces oxidation at the semiconductor and reduction at the metal, as illustrated in Figure 4b. Conversely, in the case of a p‐type semiconductor, the photoexcited holes from the VB of the p‐type semiconductor migrate and collect on the metal, thus favoring oxidation on the metal and reduction on the semiconductor, as shown in Figure 4a.

Previously, our group combined experiments with theoretical simulations to demonstrate a set of design rules and working principles in p‐type semiconductor–metal hybrid structures incorporating Cu_2_O(100)–Pd and Cu_2_O(111)–Pd heterojunctions; these devices form Schottky barriers and ohmic interfaces, respectively, that result in improved photocatalysis. The Cu_2_O(100) work function is 7.2 eV and that of Cu_2_O(111) is 4.8 eV. At the Cu_2_O(100)–Pd junction, the Pd metal work function is 1.714 eV lower than that of Cu_2_O(100), inducing migration of free electrons from Pd to Cu_2_O(100) that results in a Schottky barrier. The formation of such a barrier at the Cu_2_O(100)–Pd interface facilitates the migration of photoexcited holes to Pd metal and retains photoexcited electrons on Cu_2_O(100), which results in efficient charge separation and improved optoelectronic conversion. At the Cu_2_O(111)–Pd interface, the Pd metal work function is 2.523 eV higher than that of Cu_2_O(111), which disfavors formation of a Schottky barrier and allows ohmic contact formation. Our investigation showed that the design of the semiconductor surface facet matters both for the establishment of a Schottky barrier/ohmic contact and for spatial charge separation. By taking the advantage of Schottky barrier formation, Pd‐decorated Cu_2_O cubes at moderate Pd density produced hydrogen at a rate of 2.20 mmol g^−1^.^[^ ^36^ ^]^

Since the work function plays a critical role in determining the best type of heterojunction, tuning the work function using different approaches, such as facet selection, should provide a method of tuning charge separation. For example, Li et al.^[^ ^37^ ^]^ designed a facet‐dependent n‐type‐metal heterojunction for spatial separation of photogenerated electrons and holes using Pt, Au, and Ag metals with (010) and (110) BiVO_4_ crystal facets.^[^ ^38^ ^]^ Their results showed that metals or oxides can be deposited selectively on speciﬁc facets of BiVO_4_; different facets result in selective accumulation of electrons (holes) and adsorption of metal ions, leading to selective photodeposition and efficient charge separation.

It is universally concluded that Schottky barriers created at the interface of a semiconductor and metal allow a one‐way flow of charges that ensure efficient charge separation of photogenerated charge carriers, thus enhancing photocatalytic performance. Although this principle is well accepted for bulk heterojunctions, it may not work for nanosized ones. For bulk heterojunction materials, the Fermi level—responsible for charge migration—greatly depends on carrier concentration. However, Fermi levels of nanosized heterojunction materials are greatly affected by quantum‐size effects, surface terminations/states, lattice distortions, and impurity doping. Mechanisms behind the improved photocatalytic activity of nanosized heterojunction systems have been investigated.^[^ 39, 40 ^]^

In parallel, Yan et al. have studied the formation of nanosized Schottky or ohmic junctions that greatly improve photocatalytic activity. Their work on the nanosized ohmic junction Ag/ZnO and Schottky junction Pt/ZnO shows that the Ag/ZnO ohmic junction exhibits higher photocatalytic efficiency than the Schottky junction Pt/ZnO model system. The separation of the photogenerated charge carriers that results in improved efficiency is greatly influenced by quantum size effects and the direction of the electric fields within the semiconductor–metal interface, which together strongly influence photocatalytic efficiency.^[^ ^41^ ^]^

In contrast, the plasmonic effect occurs only under certain controlled conditions, when plasmonic metal bands are located in the visible or the near‐infrared (NIR) regions. Materials incorporating Au, Ag, or Cu—termed “plasmonic metals”—have strong plasmonic properties and have bands that are indeed located in the visible or NIR region. Importantly, the plasmonic properties of a metal are highly dependent on the size and shape of its layer, which can range from tens to hundreds of nanometers; thus, by increasing metal particle size and the dielectric properties of the surrounding medium, absorption can be shifted into the visible light region. Some other metals, such as Pt and Pd, possess very small excitation cross‐sections for surface plasmons and in small particle sizes their plasmonic bands are mainly located in the UV region.^[^ ^12^ ^]^

Plasmonic Au/TiO_2_ heterojunction photocatalysts that exhibit eﬃcient charge separation have been synthesized by Bian et al. Due to surface plasmon resonance (SPR), Au NPs layered on the basal and lateral surfaces of TiO_2_ impart a strong photoelectrochemical response in the visible light region (400–800 nm). Electrons injected from excited Au NPs (nanoparticles) on the basal surfaces of meso*‐*TiO_2_ are eﬀiciently delivered to the lateral surfaces of the crystal through the TiO_2_ nanocrystal network. This feature allows for reduced loading of the metal on the semiconductor, which is especially advantageous for NIR‐active metallic nanostructures requiring larger sizes (e.g., Au nanorods). This anisotropic electron ﬂow appreciably minimizes the recombination of electrons and holes in the Au NPs and enhances visible light photocatalytic activity by more than an order of magnitude, as compared to that of conventional Au/TiO_2_ NP systems.^[^ ^42^ ^]^

Jiang et al. studied the key role of metals in the photocatalytic activity of Au–CeO_2_ junctions. They explored the effects on the equilibrium between plasmon resonance and surface catalysis by loading various amounts and particle sizes of Au NPs on CeO_2_. Photoexcitation and surface catalysis vary inversely with Au NP size, but both NP loading and size determine the ﬁnal photocatalytic performance of propylene oxidation under visible (>420 nm) light illumination. Increasing Au loading seems to promote photoabsorption, charge separation, and resonant energy transfer due to enhanced Au SPR. However, increased Au particle size leads to saturation and also decreases the number of exposed active sites that can adsorb reactant species. In addition, large Au NPs (>10 nm) demonstrate distinct passivity toward O_2_ dissociation and activation. In general, this investigation shows that the design of eﬃcient metal−semiconductor systems for ideal solar energy conversion requires medium‐sized particles (6–12 nm) for optimizing the plasmonic and catalytic properties of metallic nanostructures.^[^ ^43^ ^]^

Despite that so many different junction designs have been investigated, the overall principles are more or less similar to those discussed earlier. Representative works on Schottky/ohmic junctions and plasmonic effects are summarized in **Table** **2**.

**Table 2:** Summary of representative semiconductor–metal junctions

Model/sample	Junction type	Oxidation site	Reduction site	Charge migration direction across the junction	Absorbance	Application	References
Pt–PbS	Schottky	PbS	Pt	PbS 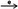 Pt	532 nm	Water splitting	[224]
Co–TiO_2_	Schottky	TiO_2_	Co	TiO_2_ 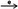 Co	–	Hydrogen evolution	[225]
Au–Bi_2_S_3_	Schottky/Plasmon	Bi_2_S_3_	Au	Bi_2_S_3_ 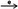 Au	560 nm	Degradation of MB	[226]
Pd–SiC	Schottky	SiC	Pd	SiC 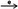 Pd	530 nm	Hydrogenation of furan derivatives	[227]
Ag–ZnO	Plasmon/Schottky	ZnO	Ag	Ag 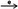 ZnO	400 nm	Degradation of RhB	[228]
Ag–TiO_2_	Schottky	TiO_2_	Ag	TiO_2_ 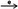 Ag	380–500 nm	Degradation of RhB	[229]
Au–TiO_2_	Plasmon/Schottky	TiO_2_	Au	Au 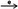 TiO_2_	540–550 nm	Ethanol–water	[230]
Au–ZnO	Schottky	ZnO	Au	ZnO 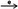 Au	350 nm	Degradation of RhB	[231]
Au–TiO_2_	Schottky/Plasmon	Au	TiO_2_	Au 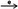 TiO_2_	*λ* > 410 nm	Degradation MO	[232]
W–WO_3_	Ohmic	WO_3_	W	WO_3_ 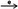 W	350–400 nm	Degradation of gaseous acetaldehyde	[233]
Au–BiVO_4_	Plasmon/Schottky	Au	BiVO_4_	Au 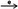 BiVO_4_	*λ* > 420 nm	Dye degradation and water oxidation	[234]
Ni–TiO_2_	Plasmon	Ni	TiO_2_	Ni 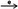 TiO_2_	495 nm < *λ* < 800 nm	Degradation of MB	[235]
Au–TiO_2_	Plasmon	Au	TiO_2_	Au 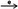 TiO_2_	*λ* > 515 nm	Water splitting	[236]
Ag–ZnO	Plasmon	Ag	ZnO	Ag 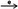 ZnO	438 nm	Degradation of MO	[237]
Ag–TiO_2_	Plasmon	Ag	TiO_2_	Ag 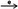 TiO_2_	553 nm	Degradation of RhB	[238]
Au–SnO_2_	Plasmon	SnO_2_	Au	Au 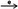 SnO_2_	550 nm	Degradation of RhB	[239]
Au–TiO_2_	Plasmon	Au	TiO_2_	Au 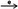 TiO_2_	550 nm	Decomposition of MB	[240]
M(Au, Ag, Cu) –TiO_2_	Plasmon	M	TiO_2_	M 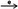 TiO_2_	Au (521 nm), Ag (540 nm) and Cu (800 nm)	Photo‐oxidation of benzaldehyde and nitrobenzaldehyde	[241]
Bi–TiO_2_	Plasmon	Bi	TiO_2_	Bi 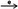 TiO_2_	λ > 420 nm	Removal of ppb‐level NO in air	[242]
Au–WO_3_	Plasmon	Au	WO_3_	Au 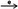 WO_3_	450–700 nm	RhB photodegradation	[243]
Au–TiO_2_	Plasmon	Au	TiO_2_	Au 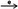 TiO_2_	*λ* > 450 nm	Oxidation of 1‐phenylethanol	[244]
Ag–TiO_2_	Plasmon	Ag	TiO_2_	Ag 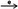 TiO_2_	450–700 nm	Water splitting and methanol oxidation	[245]
Ag–TiO_2_	Plasmon	Ag	TiO_2_	Ag 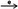 TiO_2_	*λ* = 400–750 nm	MB photodegradation	[246]
Au–CeO_2_	Plasmon	Au	CeO_2_	Au 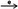 CeO_2_	*λ* > 520 nm	Aerobic oxidations of propylene	[43]
Au–TiO_2_	Plasmon	Au	TiO_2_	Au 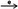 TiO_2_	548 nm	MB, RhB degradation	[42]
Au–TiO_2_	Plasmon	Au	TiO_2_	Au 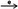 TiO_2_	Visible light	Degradation MB	[247]
Au–CdSe	Plasmon	Au	CdSe	Au 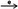 CdSe	*λ* > 700 nm	Hydrogen generation	[248]

Even though a great deal of effort has been exerted to understand the selective steering of charges at s–m junctions, it is poorly known how this weakens the redox capability of high‐energy electrons and holes at the reaction site. Apparently, charge separation and transfer works at the expense of the redox ability of charge carriers, and energy is lost during their transfer from semiconductor to metal due to the difference between the *E*_fm_ energy levels and the CB of an n‐type semiconductor (or the VB of a p‐type semiconductor). Still, a significant amount of leading‐edge research is being undertaken to overcome the challenges of attaining the efficient charge separation required for improved optoelectronic conversion. Semiconductor–semiconductor–metal and semiconductor–metal–semiconductor heterojunctions are other well‐studied heterojunctions for minimizing the drawbacks of p–n and semiconductor–metal heterojunctions.

## 5 All‐Solid‐State Ternary Heterojunctions (Z‐Schemes)

It has been a long‐standing challenge to realize the high degree of charge separation in semiconductor‐based heterojunction photocatalysts necessary for efficient optoelectronic conversion. In contrast to Type‐II and Type‐III semiconductor–semiconductor (s1–s2) junctions that steer charge flow favorably and ensure charge separation onto separate semiconductors, most s1–s2 heterojunctions fail due to their straddling bandgaps (Type I) (Figure 2a) in which both photogenerated electrons and holes are deposited in the same semiconductor with a small bandgap; this circumstance results in high recombination.

**Figure 5:**
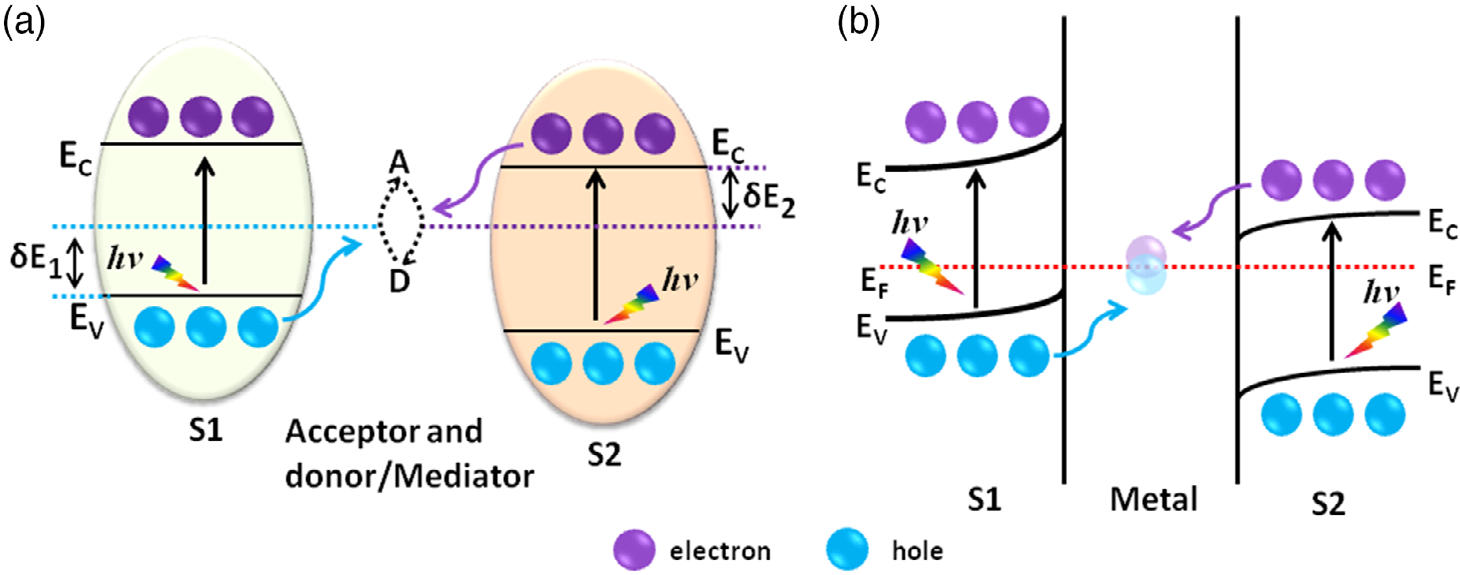
a) Schematic of band alignments and charge flows in the Z‐scheme. δ*E*_1_ (δ*E*_2_) denotes the energy barrier for diffusion of high energy holes (electrons) across the interface or is the relative potential between VB of S1 (CB of S2) and the oxidation (reduction) potential of acceptor (A) and donor (D)‐species. b) Schematic showing the band bending and charge separation mechanism in an all‐solid‐state S1–m–S2 Z‐scheme heterojunction.

Inspired by the natural process of photosynthesis in green plants, the Z‐scheme has become prominent recently as an additional model system for water splitting. This scheme includes two different semiconductors (s1 and s2) and an appropriately reversible acceptor/donor pair (AD‐species) and carries out two half‐reactions on the corresponding surfaces of the semiconductors, as shown in **Figure** **5**a.

In other words, the Z‐scheme guarantees that each of two half‐reactions can be realized on one of two spatially separated semiconductors possessing moderate *E*_*g*_. Essentially, photons with moderate energy can drive this system, which means that the visible light portion of the solar spectrum can be utilized. Importantly, AD‐species (such as Fe^3+^/Fe^2+^ and IO_3_^−^/I^−^) can be interposed to effect the separation of charge carriers formed by the consumption of energetic holes from s1 and electrons from s2. There are also some Z‐scheme systems without AD‐species in which two different semiconductors are in direct contact with each other, and in which energetic holes from the CB of s1 recombine with high‐energy electrons from the VB of s2 at the interface junction. Unfortunately, there remain drawbacks restricting further photocatalytic enhancement of Z‐scheme systems. For instance, there is no driving force to accelerate the process of reversible transition of redox mediators for realizing effective spatial separation of charges or for promoting the recombination of e–h, especially for high energy holes with low migration rates in s1. Hence, this architecture relies strongly on the band positions between the VB of s1 and the CB of s2 relative to the redox potential of the AD species (*E*_1_ and *E*_2_, respectively); this places restrictions on which combinations of semiconductors will work synergistically to create efficient photocatalytic heterostructures. A detailed tutorial and reviews of such conventional Z‐scheme systems exist.^[^ 2, 44, 45, 46 ^]^ Another well‐known Z‐scheme heterojunction is an all‐solid‐state semiconductor–metal–semiconductor/semiconductor–semiconductor–metal ternary Z‐scheme that pays considerable attention to overcoming the aforementioned drawbacks and puts forth a solution to the two central issues of charge transport—the steering of charge flows across the interface and the assurance of charge separation—so as to improve photocatalytic efficiency (shown schematically in Figure 5b). This architecture alleviates the problem of band edge position mismatch between two semiconductors by aligning their Fermi levels and shifting the bands up or down in a Z‐scheme while also minimizing lattice mismatch and transforming the Type‐I and Type‐III p–n junctions into Type II—thus making efficient charge separation more favorable and hence improving photocatalysis. All‐solid‐state Z‐scheme heterojunction designs have been reviewed specifically.^[^ 28, 46, 47, 48, 49, 50 ^]^ Here, we focus mainly on the recent advances and progress in these all‐solid‐state Z‐scheme designs.

**Figure 6:**
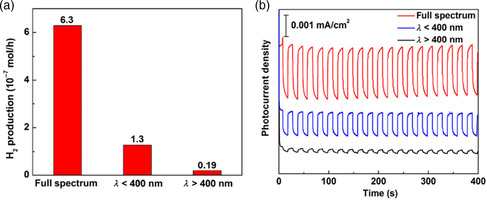
a) Average rates of photocatalytic hydrogen production and b) photocurrents versus time (*I*–*t*) curves of Ag_2_S–(Ag)–TiO_2_ hybrid structures under various light illumination conditions (full spectrum, *λ* < 400 nm and *λ* > 400 nm). a,b) Reproduced with permission.^[^ ^51^ ^]^ Copyright 2015, Tsinghua University Press and Springer‐Verlag Berlin Heidelberg.

In 2015, Li et al. from our group used a combination of theory and experiment to achieve a perfect Z‐scheme by properly aligning the bandgap of Ag_2_S in a TiO_2_–Ag–Ag_2_S heterojunction. The work function difference between Ag and Ag_2_S caused free electrons to flow from the low work function (high Fermi level) of Ag to the high work function (low Fermi level) of Ag_2_S until their Fermi levels equilibrated, resulting in an upshift and downward band bending. As a consequence, the TiO_2_–Ag–Ag_2_S heterostructure aligned to form a perfect Z‐scheme. Upon exposure of the heterojunction to light, high energy photoexcited holes and electrons residing on TiO_2_ and Ag_2_S favor oxidation and reduction, respectively, on the separate semiconductors, whereas low‐energy electrons and holes are confined to the intermediate Ag metal. The rate of hydrogen generation by this system under full solar insolation is 6.3 μmol h^−1^—higher than the sum of *λ* < 400 nm (1.3 μ molh^−1^) and *λ* > 400 nm (0.19 μmol h^−1^), as shown in **Figure** **6**.^[^ ^51^ ^]^

The performance enhancement suggests that the TiO_2_–Ag–Ag_2_S Z‐scheme substantially improved the charge generation and charge separation processes under full spectrum illumination. Average lifetime analysis of the photogenerated carriers from open‐circuit voltage decay measurements under full light spectrum illumination confirms the improvement in charge separation. The carrier lifetime of TiO_2_–Ag–Ag_2_S is prolonged, demonstrating the key role trace Ag plays in the Z‐scheme architecture.

Zhuang et al. from our group, successfully synthesized ZnS–CdS binary and ZnS–(CdS–Au, Pt, and Pd) ternary heterojunctions; these nanosystems justify and confirm that a transition from Type I to Type II is possible and established a promising approach for improving the efficiency of such systems. The work function difference (*W*_CdS_ > *W*_m_) of the ZnS–(CdS–Au, Pt, and Pd) heterojunction allows free electrons to flow from metal to CdS until the Fermi levels align and results in the upshifting and downward band bending of CdS. When the heterostructure is exposed to light, high energy photogenerated electrons migrate to ZnS while photogenerated holes migrate to the CdS/metal interface, ensuring efficient charge separation. Activities of these nanosystems, measured as the rate of hydrogen evolution, are 13.5, 36.5, 83.5, and 101 μmol h^−1^ for ZnS–CdS, ZnS–(CdS–Au), ZnS–(CdS–Pd), and ZnS–(CdS–Pt), respectively.^[^ ^52^ ^]^

Li et al. compared work functions of the CdS–Au–WO_3_ and CdS–Pt–WO_3_ heterostructures, determining that the sequential work functions of these two heterostructures are 4.9 < 5.0 < 5.05 and 4.9 < 5.2 > 5.05 eV, respectively. These results imply that the Fermi level of CdS is higher than that of either Au or Pt and that the Fermi level of WO_3_ is between the levels of these two. In this system, free electrons transfer from CdS to Au (or Pt), from Au to WO_3_, and also from WO_3_ to Pt; this results in the formation of depletion and accumulation layers at the CdS/Au and Au/WO_3_ interfaces, respectively, and depletion layers on both sides of the CdS/Pt and Pt/WO_3_ interfaces.

When the CdS–Au–WO_3_ heterojunction is exposed to light, photoinduced electrons from WO_3_ transfer to the intermediate Au layer, and holes are created at the WO_3_ semiconductor layer under the influence of an interfacial electric field, favoring oxidation. At the CdS–Au interface, photoinduced holes migrate to the intermediate Au layer and electrons collect at CdS, where reduction can then take place. The CdS–Pt–WO_3_ heterojunction functions similarly.

The finding shows explicitly that work function differences result in a lower Schottky barrier height (*W*_CdS_−*W*_Au_ < *W*_CdS_–*W*_Pt_) at the Au/CdS interface than at Pt/CdS; thus charge carrier transfer in the WO_3_/Au/CdS heterostructure is smoother, and H_2_ evolution efficiency better than in WO_3_/Pt/CdS. This work demonstrates that modulation of the intermediate metal work function plays a crucial role in Z‐scheme photocatalysis.^[^ ^53^ ^]^

As promising types of photocatalysts, generally, all Z‐scheme (i.e., including all‐solid‐state Z‐scheme and p–n junctions) heterojunction designs have been proposed to harvest energy over a broad solar spectral range by interfacing two semiconductors with a staggered bandgap. Their utilization is, however, often limited by the difficulty of achieving selective collection of low energy photogenerated charges at the interface while excluding high energy ones. Building on semiconductor–metal, p–n, and Z‐scheme designs, we and other groups have performed experimental and theoretical first‐principles investigations on energy‐dependent Z‐scheme designs of p‐type–metal–n‐type and n‐type–metal–p‐type heterostructures, which hold the promise of being able to steer charges selectively to the intermediate metal for attaining more effective charge separation. Representative summary of semiconductor–semiconductor–metal and semiconductor–metal–semiconductor photocatalyst junction studies are presented in **Table** **3**.

**Table 3:** Summary of representative semiconductor–semiconductor–metal and semiconductor–metal–semiconductor photocatalyst junction studies

Sample/model	Heterojunction type	Oxidation site	Reduction site	Charge‐transfer direction across the junction	Activity test/application	References
Ag_2_O‐Fe‐TiO_2_	p‐S1‐m‐n‐S2	TiO_2_	Ag_2_O	TiO_2_ 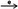 Fe, Ag_2_O 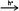 Fe	CO_2_ Conversion	[249]
BiOBr–Ag–AgBr	p‐S1‐m‐n‐S2	BiOBr	AgBr	BiOBr 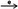 Ag, AgBr 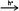 Ag	MO degradation	[250]
Ag_3_PO_4_–Ag–Bi_2_MoO_6_	p‐S1‐m‐n‐S2	Ag_3_PO_4_	Bi_2_MoO_6_	Ag_3_PO_4_ 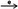 Ag, Bi_2_MoO_6_ 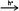 Ag	RhB degradation	[251]
TiO_2_–Pt–CdS	n‐S1‐m‐n‐S2	TiO_2_	CdS	TiO_2_ 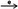 Pt, CdS 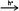 Pt	CO_2_ Photoreduction	[252]
AgCl–Ag–BiOCl	n‐S1‐m‐p‐S2	AgCl	BiOCl	Ag 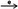 BiOCl, Ag 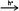 AgCl	MO Degradation	[253]
CdS–Au–ZnO	n‐S1‐m‐n‐S2	ZnO	CdS	ZnO 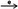 Au, CdS 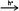 Au	H_2_ generation	[254]
CdS–Au–TiO_2_	n‐S1‐m‐n‐S2	TiO_2_	CdS	TiO_2_ 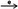 Au, Au 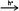 TiO_2_	MV^2+^reduction	[255]
CdS–Au–BiOCl	n‐S1‐m‐p‐S2	BiOCl	CdS	BiOCl 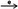 Au, CdS 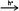 Au	MO, RhB, and Phenol Degradation	[256]
BiVO_4_–Au–Cu_2_O	n‐S1‐m‐p‐S2	BiVO_4_	Cu_2_O	BiVO_4_ 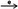 Au, Cu_2_O 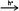 Au	CO_2_ Photoreduction	[257]
ZnO–Pt–CdS	n‐S1‐m‐n‐S2	CdS	Pt	CdS 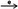 ZnO, ZnO 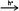 Pt	H_2_ generation	[258]
CdS–Ag–Bi_2_MoO_6_	n‐S1‐m‐n‐S2	Bi_2_MoO_6_	CdS	Bi_2_MoO_6_ 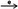 Ag, CdS 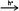 Ag	RhB degradation	[259]
BiVO_4_–Ag–Cu_2_O	n‐S1‐m‐p‐S2	BiVO_4_	Cu_2_O	BiVO_4_ 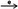 Ag, Ag 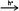 Cu_2_O	TC degradation	[260]
WO_3_–Ag–AgCl	n‐S1‐m‐n‐S2	WO_3_	AgCl	Ag 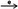 AgCl, Ag 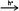 WO_3_	RhB degradation	[261]
In_2_S_3_–Au–BiVO_4_	n‐S1‐m‐n‐S2	BiVO_4_	In_2_S_3_	BiVO_4_ 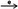 Au, In_2_S_3_ 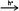 Au	RhB and phenol Degradation	[262]
TiO_2_–Pt–SnO_2_	n‐S1‐m‐n‐S2	SnO_2_	TiO_2_	SnO_2_ 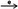 Pt, TiO_2_ 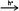 Pt	MB degradation	[263]
CdS–Pt–Mo_2_C	n‐S1‐m‐p‐S2	CdS	Pt	CdS 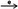 Mo_2_C, Mo_2_C 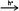 Pt	H_2_ generation	[264]
α‐Fe_2_O_3_–Ag–AgCl	n‐S1‐m‐p‐S2	a‐Fe_2_O_3_	AgCl	AgCl 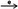 Ag, AgCl 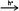 α‐Fe_2_O_3_	RhB degradation	[265]
Ag_3_PO_4_–Ag–SiC	n‐S1‐m‐n‐S2	Ag_3_PO_4_	SiC	Ag_3_PO_4_ 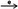 Ag, SiC 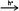 Ag	MO and Phenol degradation	[266]
α/β‐Bi_2_O_3_–Ag–AgCl	n‐S1‐m‐n‐S2	Bi_2_O_3_	AgCl	Bi_2_O_3_ 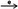 Ag, Ag 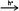 AgCl	RhB and acid orange dyes degradation	[267]
CdS–Au–WO_3_	n‐S1‐m‐n‐S2	WO_3_	CdS	WO_3_ 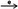 Au, Au 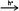 CdS	Hydrogen and oxygen evolution	[268]
Pt–CeO_2_–ZnO	m‐n‐S1‐n‐S2	Pt	CeO_2_	CeO_2_ 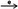 ZnO, ZnO 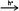 Pt, ZnO 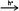 CeO_2_	Phenol degradation	[269]
Au–CuO–Co_3_O_4_	m‐p‐S1‐p‐S2	CuO,Co_3_O_4_	–	CuO 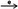 Au, Co_3_O_4_ 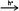 Au	O_2_ evolution	[270]
Ag–AgCl–BiPO_4_	m‐n‐S1‐n‐S2	Ag	BiPO_4_	Ag 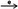 AgCl, AgCl 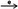 BiPO_4,_ BiPO_4_ 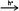 AgCl	RhB degradation	[271]
Ag–AgVO_3_–BiOCl	m‐n‐S1‐n‐S2	AgVO_3_	BiOCl	AgVO_3_ 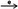 Ag, AgVO_3_ 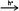 BiOCl	MB degradation	[272]
Au–ZnO–TiO_2_	m‐n‐S1‐n‐S2	ZnO	Au	Au 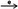 TiO_2_, ZnO 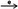 TiO_2_, TiO_2_ 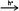 ZnO	Photo‐oxidation of phenol and 4‐chlorophenol	[273]
Ag–AgBr–InVO_4_	m‐n‐S1‐n‐S2	InVO_4_	AgBr	InVO_4_ 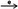 AgBr, Ag 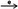 AgBr, AgBr 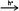 InVO_4_	RhB degradation	[274]
Ag–TiO_2_–ZnO	m‐n‐S1‐n‐S2	ZnO	Ag	ZnO 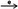 TiO_2_, TiO_2_ 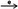 Ag, TiO_2_ 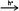 ZnO	Phenol degradation	[275]
Ag–SnS–TiO_2_	m‐n‐S1‐n‐S2	SnS	Ag	SnS 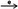 Ag, TiO_2_ 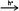 Ag	RhB and MB degradation	[276]
Ag–AgBr–Ag_3_VO_4_	m‐n‐S1‐n‐S2	AgBr‐	Ag_3_VO_4_	Ag 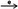 AgBr, Ag 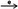 BrAg_3_VO_4_, Ag_3_VO_4_ 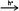 AgBr	MO degradation	[277]
Ag–Ag_2_O–BiOCl	m‐p‐S1‐p‐S2	Ag_2_O	BiOCl	Ag_2_O 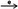 Ag, BiOCl 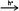 Ag	RhB degradation	[278]
Ag–Ag_2_S–CuS	m‐n‐S1‐p‐S2	Ag_2_S	Ag	CuS 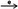 Ag, Ag_2_S 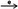 Ag, CuS 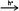 Ag_2_S	2,4‐Dichlorophenol degradation	[279]
Ag–AgCl–ZnO	m‐n‐S1‐n‐S2	AgCl	ZnO	AgCl 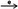 ZnO, Ag 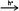 ZnO	RhB and MO degradation	[280]
Cu–Cu_2_O–ZnO	m‐p‐S1‐n‐S2	Cu_2_O	ZnO	Cu 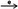 Cu_2_O, Cu_2_O 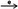 ZnO, ZnO 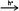 Cu_2_O	H_2_ generation	[281]
Pt–In_2_O_3_–TiO_2_	m‐n‐S1‐n‐S2	In_2_O_3_	TiO_2_	Pt 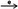 In_2_O_3_, In_2_O_3_ 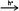 TiO_2_	CR degradation and H_2_ generation	[282]
Pt–In_2_O_3_–TiO_2_	m‐n‐S1‐n‐S2	In_2_O_3_	Pt	In_2_O_3_ 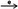 TiO_2_, TiO_2_ 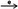 Pt, TiO_2_ 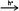 In_2_O_3_	RhB degradation	[283]
Ag‐AgBr‐Bi_2_MoO_6_	m‐n‐S1‐n‐S2	AgBr	Bi_2_MoO_6_	Ag 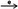 AgBr, Ag 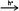 Bi_2_MoO_6_	MB degradation	[284]
Au–TiO_2_–SnO_2_	m‐n‐S1‐n‐S2	TiO_2_	SnO_2_	Au 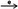 TiO_2_, Au 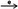 SnO_2_, TiO_2_ 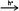 SnO_2_	RhB degradation	[285]
TiO_2_–Fe_2_O_3_–Cu	n‐S1‐n‐S2‐m	TiO_2_	Fe_2_O_3_	TiO_2_ 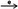 Fe_2_O_3_, Fe_2_O_3_ 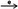 Cu, Fe_2_O_3_ 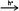 TiO_2_	MO degradation	[286]
Cu–Cu_2_O–ZnO	m‐p‐S1‐n‐S2	Cu_2_O	ZnO	Cu 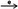 Cu_2_O, Cu 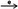 ZnO, Cu_2_O 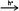 ZnO	MB degradation	[287]
Pt–CdS–h–BiOBr	m‐n‐S1‐p‐S2	BiOBr	CdS	Pt 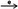 h‐BiOBr, h‐BiOBr 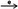 CdS, CdS 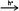 h‐BiOBr	MB degradation	[288]
Ag–AgBr–TiO_2_	m‐n‐S1‐n‐S2	AgBr	TiO_2_	Ag 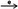 AgBr, Ag 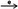 BrTiO_2_, TiO_2_ 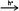 AgBr	MB degradation	[289]
Ag–SrTiO_3_–TiO_2_	m‐n‐S1‐n‐S2	SrTiO_3_	TiO_2_,Ag	SrTiO_3_ 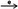 Ag, SrTiO_3_ 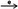 TiO_2_, TiO_2_ 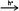 SrTiO_3_	RhB degradation	[290]
Cu–SrTiO_3_–TiO_2_–Cu	m‐n‐S1‐n‐S2‐m	Cu	Cu	SrTiO_3_ 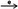 TiO_2_, TiO_2_ 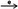 Cu, TiO_2_ 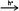 SrTiO_3_, SrTiO_3_ 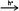 Cu	H_2_ generation	[291]
Fe_3_O_4_–TiO_2_–Ag	n‐S1‐n‐S2‐m	Ag	Fe_3_O_4_	Ag 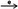 TiO_2_, TiO_2_ 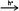 Fe_3_O_4_	AMP degradation	[292]
CuS–TiO_2_–Pt	p‐S1‐n‐S2‐m	CuS	Pt	TiO_2_ 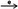 Pt, TiO_2_ 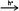 CuS	H_2_ production	[293]
In_2_O_3_–ZnO–Ag	n‐S1‐n‐S2‐m	In_2_O_3_	ZnO	In_2_O_3_ 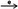 ZnO, Ag 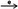 ZnO, ZnO 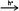 Ag nanowire	MO and 4‐nitrophenol degradation	[294]
Ag_3_VO_4_–Ag_3_PO_4_–Ag	p‐S1‐p‐S2‐m	Ag_3_VO_4_	Ag	Ag_3_VO_4_ 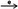 Ag_3_PO_4_, Ag_3_PO_4_ 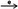 Ag, Ag_3_PO_4_ 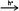 Ag_3_VO_4_	Acid blue 92 (AB92) degradation	[295]
NiO–ZnO–Au	p‐S1‐n‐S2‐m	ZnO	NiO, Au	ZnO 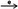 Au, ZnO 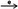 NiO, NiO 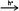 ZnO	RhB degradation	[296]
NiO–ZnO–Pt	p‐S1‐n‐S2‐m	ZnO	NiO, Pt	ZnO 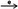 Pt, ZnO 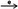 NiO, NiO 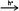 ZnO	MO degradation	[297]
MoS_2_–TiO_2_–Au	p‐S1‐n‐S2‐m	MoS_2_	TiO_2_	Au 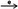 TiO_2_, Au 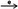 MoS_2_, MoS_2_ 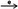 TiO_2,_ TiO_2,_ 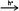 MoS_2_	Current density and H_2_ generation	[298]

**Figure 7:**
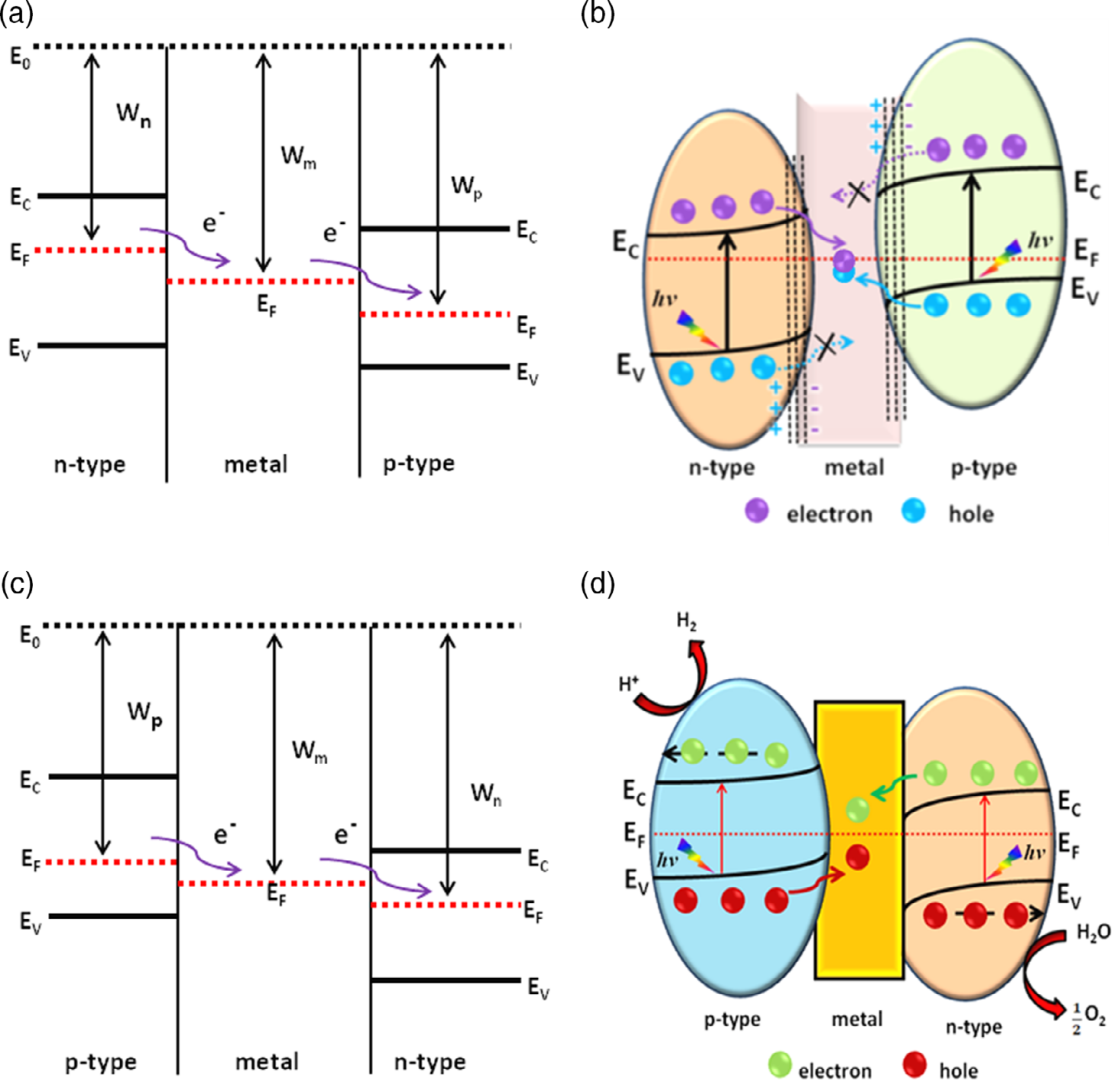
Energy‐dependent Z‐scheme designs of n‐type–metal–p‐type Schottky junction and p‐type–metal–n‐type ohmic junction models in which: a) An energy‐dependent Z‐scheme n–m–p design allows free electron migration from n‐type to metal and then to p‐type until Fermi levels are equilibrated. b) Upon illumination low‐energy photoexcited electrons from the n‐type layer and holes from p‐type are transferred to the intermediate metal layer while high‐energy electrons are confined to their semiconductors. a,b) Reproduced with permission.^[^ ^54^ ^]^ Copyright 2020, American Chemical Society. c) An energy‐dependent Z‐scheme p–m–n design allows free electrons to migrate from p‐type to metal to n‐type until the Fermi levels are equilibrated. d) Upon illumination, low‐energy photoexcited holes from the p‐type layer and electrons from n‐type are transferred to the intermediate metal and high‐energy electrons are confined to the individual semiconductors. c,d) Reproduced with permission.^[^ ^55^ ^]^ Copyright 2019, Royal Society of Chemistry.

Recently, our research group has proposed a promising ternary energy‐dependent Z‐scheme incorporating both an n‐type semiconductor–metal–p‐type–semiconductor and a p‐type semiconductor–metal–n‐type semiconductor heterojunction photocatalyst using first‐principles calculations. This design features a work function cascade with *W*_n_ < *W*_m_ < *W*_p_ and *W*_p_ < *W*_m_ < *W*_n_ for the two heterojunctions, respectively (**Figure** **7**). These systems are designed to steer charge flow and enhance effective charge separation by combining the merits of traditional Z‐scheme, p–n, and s–m systems so that they work synergistically to realize high photocatalytic performance. The intermediate metal not only lowers the lattice matching and band alignment requirements between the two semiconductors but also induces band bending at the p–m and m–n interfaces via charge migration that lines up the Fermi levels. Such band bending enables selective steering of low energy charges from the two semiconductors to the intermediate metal while confining high‐energy electrons and holes to the individual p‐type and n‐type semiconductors, respectively, so as to enable oxidation and reduction reactions to take place.^[^ 54, 55 ^]^

A combined experimental and theoretical study of an n‐type–metal–p‐type TiO_2_–Pd–Cu_2_O Z‐scheme has been performed recently by Ye et al. Their design achieved improved photocatalysis by interfacing n‐type TiO_2_(001) and p‐type Cu_2_O(100) facets with a layer of Pd metal placed between them; this design produced the desired bandgap alignment for inducing migration of photoexcited electrons from TiO_2_(001) to Pd and holes from Cu_2_O(100) to Pd. The TiO_2_(001) < Pd < Cu_2_O(100) work functions ordering allows free electrons to flow from TiO_2_(001) to Pd and from Pd to Cu_2_O(100) and equilibrates the Fermi levels. Upon light illumination, this facet hybrid junction facilitates low energy electron transfer from the TiO_2_(001) facet to the intermediate Pd layer and hole migration from Cu_2_O(100) to Pd; good charge separation is produced by confining high energy holes at TiO_2_(001)—enabling oxidation—and high‐energy electrons at Cu_2_O(100)—enabling reduction. This hybrid TiO_2_(001) < Pd < Cu_2_O(100) design has 1.37–3.12 times higher photocurrent density and 1.22–2.06‐fold higher phenol degradation efficiency than another three‐hybrid design that also incorporates a TiO_2_(101) facet or Cu_2_O(111) facet in contact with Pd at an interface.^[^ ^56^ ^]^

Recently, an interesting n–metal–p Janus plasmonic heteronanocrystals of Au/(PbS‐CdS) have been synthesized by Wan et al. The construction of these heterojunctions investigates that hot plasmonic electrons and holes collected simultaneously on individual semiconductors. The minimal Schottky barrier and ohmic contact created at the Au–CdS and Au–PbS interfaces enhance efficient separation of plasmonic electrons and smooth transfer of hot plasmonic holes, respectively. The result shows an extended lifetime of the charge separated state, and superior in photocatalytic CO_2_ performance reduction.^[^ ^57^ ^]^

The extension of ternary heterojunction designs to include an energy‐dependent Z‐scheme p–m–n and n–m–p heterojunction that can steer charges selectively across the interface presents a better opportunity to achieve good charge separation and thereby enhance photocatalytic activity. The inner electric field established at the junction assisted by band bending drives low energy holes and electrons to the intermediary metal and thereby lowers the probability of undesirable electron–hole pair recombination and keeps high‐energy electrons and holes apart by confining them to the individual semiconductors, finally resulting in good charge separation. The intermediary metal not only serves as a center of recombination but also equilibrates the Fermi levels across the interface and minimizes lattice mismatch between the two semiconductors. In general, this p–m–n and n–m–p strategy may, thus, pave the way for making more efficient Z‐scheme photocatalysts for practical applications. Our extension of this design that utilizes thin layers in an energy‐dependent Z‐scheme, the Cu_2_S–Pt–WO_3_ (p‐type–metal–n‐type) heterojunction, permits the exploration of an alternative way of realizing efficient charge separation. This system demonstrates increased charge flow across the junction compared to its bulk counterpart and exhibits a higher electron density on each surface that should produce enhanced optoelectronic conversion.^[^ ^58^ ^]^

## 6 Semiconductor–Graphene Heterojunctions

Until now, achieving good charge separation and absorption of broad‐spectrum visible light either in a single semiconductor or by the interfacing of two semiconductors either alone or with metal is a challenge that has prevented their practical application. Shifting optical absorption from UV to the visible light region to maximize the quantum efficiency of single semiconductors in combination with graphene has emerged as a new prospect. Graphene is a well‐known material that possesses high surface area, high conductivity, and good adsorptive properties; these lead to improved accumulation of charge and effective charge separation.^[^ 20, 59, 60, 61, 62, 63, 64 ^]^

Williams et al. synthesized a photoactive graphene–TiO_2_ nanocomposite with graphene oxide (GO) suspended in ethanol. Upon UV light irradiation, photoexcited electrons transferred from TiO_2_ to GO; this was confirmed by the reduction accompanying changes in the absorption of the GO, as evidenced by the shift in color of the suspension from brown to black. No significant change was observed upon UV light illumination when TiO_2_ was excluded from the solution, confirming that surface electrons migrate from TiO_2_ and reduce GO.^[^ ^65^ ^]^ Similar studies on graphene–TiO_2_ photocatalysis using different experimental methods show improved pollutant degradation and hydrogen production.^[^ 66, 67, 68, 69, 70, 71, 72 ^]^

**Figure 8:**
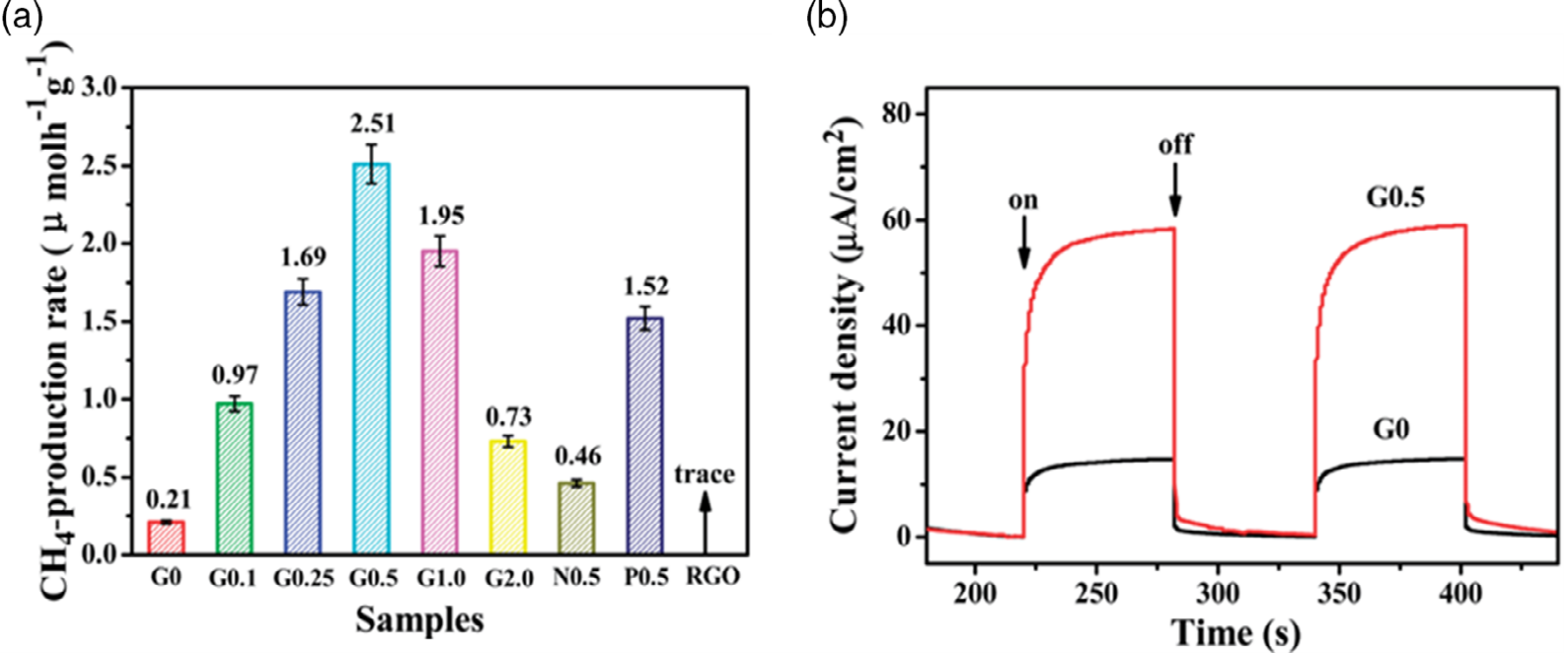
a) Comparison of photocatalytic CH_4_ production rates of G0, G0.1, G0.25, G0.5, G1.0, G2.0, N0.5, P0.5, and RGO samples under visible light irradiation. b) Transient photocurrent responses of the G0 and G0.5 samples in 0.4 m Na_2_SO_4_ aqueous solution under visible light (*λ* = 420 nm) irradiation. a,b) Reproduced with permission.^[^ ^73^ ^]^ Copyright 2014, Royal Society of Chemistry.

Yu et al. successfully synthesized RGO–CdS nanorod composite by a one‐step microwave‐assisted hydrothermal process for carrying out the photocatalytic reduction of CO_2_ to CH_4_. Their analysis revealed that the RGO–CdS nanorod junction at 0.5 wt% of RGO achieved a high production rate (2.51 μmol h^−1^ g^−1^), 10 times greater than that of pristine CdS nanorods (**Figure** **8**).

The high production rate is due to the presence of RGO that acts as an acceptor of photogenerated electrons from CdS upon irradiation.^[^ ^73^ ^]^ Similar metal‐free RGO‐Ss, such as Ag_2_CrO_4_–GO,^[^ ^74^ ^]^ TiO_2_–S/rGO,^[^ ^75^ ^]^ and GO/CuFe_2_O_4_,^[^ ^76^ ^]^ have been investigated as materials that can replace expensive noble metals with carbon, and ultimately enhance the photocatalytic activity of single semiconductors.

## 7 Semiconductor (S)–Graphitic Carbon Nitride (C_3_N_4_) Heterojunctions

Recently, other heterojunctions composed of S–C_3_N_4_ have been widely investigated by many researchers for optimizing single semiconductor photoelectronic conversion. C_3_N_4_ is inexpensive, harmless, and has a narrow bandgap of 2.7 eV, qualifying it for consideration as a photocatalytic material for H_2_ production, O_2_ evolution, and CO_2_ reduction upon visible light irradiation.^[^ 77, 78 ^]^

In a combined experimental and theoretical study, Yu et al. have demonstrated enhanced photocatalytic activity for selective reduction of CO_2_ to CH_3_OH on g‐C_3_N_4_–ZnO. The lower potential photogenerated electrons from the CB of ZnO recombine at a minimal rate with the higher potential VB‐excited holes of g‐C_3_N_4_ resulting in enhanced photocurrent production as confirmed experimentally. Indeed, this g‐C_3_N_4_–ZnO photocatalyst attains 2.3 times higher photocatalytic activity than does pure g‐C_3_N_4_.^[^ ^79^ ^]^

Li et al. synthesized a direct Z‐scheme g‐C_3_N_4_–TiO_2_ heterojunction that shows improved photocatalytic activity for degradation of the organic pollutant propylene under visible light irradiation. Upon light irradiation, photoexcited electrons migrate from the TiO_2_ layer and recombine with holes from g‐C_3_N_4_; this favors reduction at g‐C_3_N_4_ and oxidation at the TiO_2_ surface. This research attempts to demonstrate that a larger speciﬁc surface area and stronger UV–vis light absorption can result in improved photocatalytic activity due to increased numbers of active sites and photogenerated carriers. By considering two samples—20%g‐C_3_N_4_–TiO_2_–400 and 30%g‐C_3_N_4_–TiO_2_–600—they showed that increased light absorption does not always lead to improved photocatalytic activity. Rather, 20%g‐C_3_N_4_–TiO_2_–400, which possesses a larger specific surface area and better visible light absorption, results in lower photocatalytic activity than 30%g‐C_3_N_4_–TiO_2_–600. Thus, in addition to large surface area and broad light spectrum absorption, better charge transfer and separation are also crucial factors for enhanced photocatalysis.^[^ ^80^ ^]^

CQDs incorporating carbon nitride show interesting effects and attain improved quantum efficiency. For instance, Liu et al. synthesized a metal‐free carbon nanodot–carbon nitride (C_3_N_4_) nanocomposite for photocatalytic water splitting. Such a CQDs–C_3_N_4_ hybrid is made of low‐cost and environmentally friendly materials. This device attained quantum efficiencies of 16% for wavelength *λ* = 420 ± 20 nm, 6.29% for *λ* = 580 ± 15 nm, and 4.42% for *λ* = 600 ± 10 nm, and an overall solar energy conversion efficiency of 2.0%.^[^ ^81^ ^]^

Another facile experimental method has been developed by Guo et al. to synthesize an infrared‐responsive photocatalyst—a CQD/carbon nitride nanocomposite—at 450 °C, designed to enhance photocatalytic activity. In this hybrid photocatalyst, the CQD converts infrared to visible light, whereas the carbon nitride utilizes the visible light emitted by the CQD to degrade pollutants. This composite photocatalyst degrades MO efficiently under infrared light irradiation (λ > 800 nm).^[^ ^82^ ^]^

Recently, our group has performed a detailed theoretical study of metal‐free CQD/carbon‐nitride hybrid systems to investigate the mechanism across the interface for isolating hydrogen from oxygen in photocatalytic water splitting.

**Figure 9:**
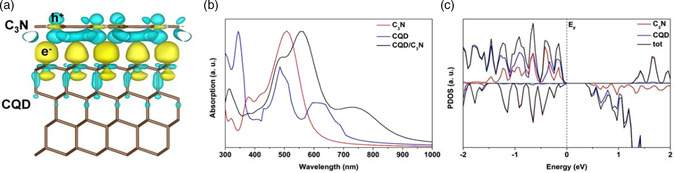
a) Differential charge distribution of the carbon quantum dot (CQD)/C_3_N structure. The yellow and blue regions represent electron and hole charge distributions, respectively, with an isosurface value of 0.0006 e Å^−3^. b) Photoabsorption spectrum of the bare C_3_N (red), CQD (blue), and CQD/C_3_N composite structures (black). c) PDOS of the CQDs/C_3_N hybrid. a–c) Reproduced with permission.^[^ ^83^ ^]^ Copyright 2018, Royal Society of Chemistry.

The work functions of C_3_N and the CQDs were calculated to be 3.25 and 4.65 eV, respectively, so that electron flow is driven from C_3_N to the CQDs. The computed CQD/C_3_N differential charges indicate that holes collect at C_3_N and electrons at the CQDs. The computed absorption coefficients, as shown in **Figure** **9**b, reveal that while bare C_3_N absorbs mainly short‐wavelength visible light (<600 nm) and CQD absorption extends to ≈700 nm, absorption of the CQDs/C_3_N composite extends to ≈900 nm.

Extended time‐dependent density functional theory (TDDFT) calculations reveal that photoexcited electrons transfer from C_3_N to CQD, which is in good agreement with ultrafast charge evolution results, which favor oxidation at C_3_N and reduction at CQD sites. This CQDs/C_3_N hybrid photocatalyst design enables the full use of visible and IR light to generate and distribute high energy holes and electrons on the C_3_N and CQDs layers. Representative works on S–g‐C_3_N_4_ photocatalyst junctions are summarized in **Table** **4**.^[^ ^83^ ^]^

**Table 4:** Summary of representative S‐g‐C_3_N_4_ photocatalyst junctions reported to date

Model/sample	Oxidation site	Reduction site	Charge‐transfer direction across the junction	Activity test/application	References
g‐C_3_N_4_/TiO_2_	TiO_2_	g‐C_3_N_4_	TiO_2_ 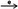 Interface, g‐C_3_N_4_ 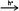 Interface	Degradation of propylene and hydrogen generation, TC, degradation of diclofenac	[299, 300]
C_3_N_4_/BiOIO_3_	BiOIO_3_	C_3_N_4_	BiOIO_3_ 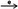 Interface, C_3_N_4_ 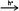 Interface	Photodegradation of MO, RhB	[301]
g‐C_3_N_4_/Bi_4_O_7_	Bi_4_O_7_	g‐C_3_N_4_	Bi_4_O_7_ 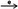 Interface, g‐C_3_N_4_ 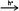 Interface	Degradation of MB, phenol, RhB, and BPA	[302]
g‐C_3_N_4_/Bi_2_MoO_6_	Bi_2_MoO_6_	g‐C_3_N_4_	Bi_2_MoO_6_ 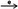 Interface, g‐C_3_N_4_ 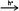 Interface	Photodegradation of MB	[303]
g‐C_3_N_4_/AgFeO_2_	AgFeO_2_	g‐C_3_N_4_	AgFeO_2_ 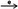 Interface, g‐C_3_N_4_ 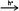 Interface	Acid red G (ARG)	[304]
g‐C_3_N_4_/Ag_2_WO_4_	Ag_2_WO_4_	g‐C_3_N_4_	Ag_2_WO_4_ 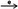 Interface, g‐C_3_N_4_ 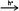 Interface	Degradation of MO	[305]
g‐C_3_N_4_/Ag_2_CrO_4_	Ag_2_CrO_4_	g‐C_3_N_4_	Ag_2_CrO_4_ 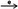 Interface, g‐C_3_N_4_ 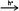 Interface	Degradation of MO	[306]
g‐C_3_N_4_/Bi_2_O_3_	Bi_2_O_3_	g‐C_3_N_4_	Bi_2_O_3_ 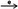 Interface, g‐C_3_N_4_ 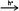 Interface	Photodegradation of MB, RhB	[307]
g‐C_3_N_4_/BiVO_4_	BiVO_4_	g‐C_3_N_4_	BiVO_4_ 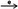 Interface, g‐C_3_N_4_ 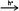 Interface	Degradation of toluene	[308]
g‐C_3_N_4_/WO_3_	WO_3_	g‐C_3_N_4_	WO_3_ 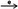 Interface, g‐C_3_N_4_ 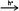 Interface	Decomposition of acetaldehyde, MB	[309, 310, 311, 312]
g‐C_3_N_4_/ZnO	ZnO	g‐C_3_N_4_	ZnO 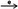 Interface, g‐C_3_N_4_ 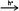 Interface	Photocatalytic CO_2_ conversion to fuel, CO_2_ reduction to CH_3_OH	[229, 313]
g‐C_3_N_4_/V_2_O_5_	V_2_O_5_	g‐C_3_N_4_	V_2_O_5_ 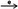 Interface, g‐C_3_N_4_ 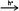 Interface	Degradation of RhB and TC	[314]
g‐C_3_N_4_/Ag_2_CrO_4_	Ag_2_CrO_4_	g‐C_3_N_4_	Ag_2_CrO_4_ 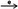 Interface, g‐C_3_N_4_ 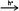 Interface	Photodegradation of MB, RhB, MO	[315, 316]
g‐C_3_N_4_/Bi_12_GeO_20_	Bi_12_GeO_20_	g‐C_3_N_4_	Bi_12_GeO_20_ 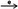 Interface, g‐C_3_N_4_ 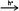 Interface	Degradation of microcystin‐LR and RhB, and for reduction of aqueous Cr(VI)	[317]
g‐C_3_N_4_/Bi_2_Sn_2_O_7_	Bi_2_Sn_2_O_7_	g‐C_3_N_4_	Bi_2_Sn_2_O_7_ 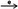 Interface, g‐C_3_N_4_ 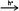 Interface	Degradation of MB and acid red 18 (AR 18)	[318]
g‐C_3_N_4_/Ag_2_CO_3_	Ag_2_CO_3_	g‐C_3_N_4_	Ag_2_CO_3_ 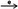 Interface, g‐C_3_N_4_ 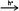 Interface	Degradation of MO	[319]
g‐C_3_N_4_/BiOI	BiOI	g‐C_3_N_4_	BiOI 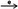 Interface, g‐C_3_N_4_ 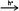 Interface	Reduction of CO_2_	[320]
g‐C_3_N_4_/Ag_3_PO_4_	Ag_3_PO_4_	g‐C_3_N_4_	Ag_3_PO_4_ 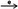 Interface, g‐C_3_N_4_ 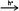 Interface	CO_2_ conversion to fuel	[321, 322]
g‐C_3_N_4_/MoO_3_	MoO_3_	g‐C_3_N_4_	MoO_3_ 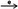 Interface, g‐C_3_N_4_ 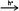 Interface	Degradation of MO	[323]
g‐C_3_N_4_/DyVO_4_	g‐C_3_N_4_	DyVO_4_	g‐C_3_N_4_ 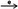 DyVO_4_, DyVO_4_ 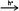 g‐C_3_N_4_	Degradation of RhB	[324]
g‐C_3_N_4_/Bi_2_WO_6_	Bi_2_WO_6_	g‐C_3_N_4_	Bi_2_WO_6_ 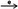 Interface, g‐C_3_N_4_ 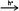 Interface	Photoreduction of CO_2_ to CO	[325]
g‐C_3_N_4_/ZrO_2_	g‐C_3_N_4_	ZrO_2_	g‐C_3_N_4_ 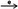 ZrO_2_, ZrO_2_ 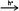 g‐C_3_N_4_	Degradation of RhB	[326]
AgCl/g‐C_3_N_4_	AgCl	g‐C_3_N_4_	AgCl 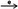 Interface, g‐C_3_N_4_ 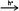 Interface	H_2_ production	[327]
BiOIO_3_/g‐C_3_N_4_	BiOIO_3_	g‐C_3_N_4_	BiOIO_3_ 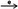 Interface, g‐C_3_N_4_ 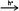 Interface	Degradation of NO	[328]
Bi_2_S_3_/g‐C_3_N_4_	Bi_2_S_3_	g‐C_3_N_4_	Bi_2_S_3_ 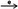 Interface, g‐C_3_N_4_ 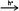 Interface	Photoreduction of CO_2_ to CO	[329]
MnCo_2_O_4_/g‐C_3_N_4_	g‐C_3_N_4_	MnCo_2_O_4_	g‐C_3_N_4_ 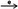 MnCo_2_O_4_, MnCo_2_O_4_ 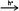 g‐C_3_N_4_	H_2_ production	[330]
WS_2_/g‐C_3_N_4_	g‐C_3_N_4_	WS_2_	g‐C_3_N_4_ 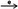 WS_2_	H_2_ production	[331]
g‐C_3_N_4_/SnS	g‐C_3_N_4_	SnS	g‐C_3_N_4_ 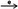 SnS, SnS 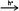 g‐C_3_N_4_	Reduction of aqueous Cr(VI)	[332]
g‐C_3_N_4_/BiOBr	g‐C_3_N_4_	BiOBr	g‐C_3_N_4_ 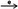 BiOBr, BiOBr 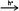 g‐C_3_N_4_	Oxidation of NO and reduction of CO_2_	[333]

## 8 Semiconductor–(RGO/Metal)–Graphitic Carbon Nitride (g‐C_3_N_4_) Heterojunctions

Recently, much effort has been devoted to the construction of all‐solid‐state Z‐scheme semiconductor–metal–g‐C_3_N_4_ and semiconductor–RGO–g‐C_3_N_4_ heterojunctions. These designs aim to minimize recombination and lattice mismatch by including metal and RGO mediators for efficient optoelectronic conversion.

Peng et al. synthesized a CdS/Au/g‐C_3_N_4_ ternary heterojunction by a facile two‐step photoreduction method; this device exhibited enhanced visible light photocatalytic performance. When the CdS/Au/g‐C_3_N_4_ heterojunction was irradiated, photogenerated electrons from CdS and holes from g‐C_3_N_4_ transferred to the intermediate metal; this process favored oxidation and reduction separately at CdS and g‐C_3_N_4_, respectively. Experimentally, the ternary CdS/Au/g‐C_3_N_4_ heterojunction exhibited better photocatalytic activity than did CdS/g‐C_3_N_4_. This may be due to the effect of the smaller sized CdS nanoparticles in the ternary than in the binary heterojunction, resulting in enhanced charge separation of photogenerated electron–hole pairs. Another possibility is that Z‐scheme formation in CdS/Au/g‐C_3_N_4_ imparts greater redox ability to excited charges than in the binary Au/g‐C_3_N_4_.^[^ ^84^ ^]^

Lately, RGO has been investigated as a mediator in semiconductor–g‐C_3_N_4_ heterojunctions. Wu et al. demonstrated an all‐solid‐state g‐C_3_N_4_–RGO–TiO_2_ Z‐scheme device that performs enhanced photocatalytic degradation of methylene blue. Upon light illumination photogenerated electrons from TiO_2_ and holes from g‐C_3_N_4_ collect in RGO, enabling oxidation at TiO_2_ and reduction at g‐C_3_N_4_. This indirect g‐C_3_N_4_–RGO–TiO_2_ Z‐scheme device exhibited a maximal degradation rate of 0.0137 min^−1^—about 4.7 and 3.2 times greater than in either pure g‐C_3_N_4_ (0.0029 min^−1^) or direct Z‐scheme g‐C_3_N_4_–TiO_2_ (0.0043 min^−1^), respectively. The improved charge transfer and separation due to incorporating RGO in this nanoheterojunction results in an enhanced reduction of oxygen at g‐C_3_N_4_ and oxidation of hydroxyl radicals at TiO_2_.^[^ ^85^ ^]^

Marchal et al. produced Au/TiO_2_‐g‐C_3_N_4_ nanocomposites with very low amounts of sacrificial agents present to split water for hydrogen production. When this heterojunction was exposed to light, photogenerated electrons from TiO_2_ and g‐C_3_N_4_ migrated to and deposited in Au nanoparticles, favoring the production of H_2_. High energy holes in the TiO_2_ and g‐C_3_N_4_ layers oxidize H_2_O and produce oxygen. This composite photocatalyst yields high H_2_ production (350 μmol h^−1^ g^−1^ catalyst) using minimal amounts of sacrificial agent (≤1 vol%); its performance exceeds that of either binary Au/TiO_2_ or Au/g‐C_3_N_4_ under solar and visible light irradiation. This enhanced performance is due to the homogeneous deposition of Au NPs onto both semiconductors, the SPR of the Au NPs, and the perfect VB and CB alignment forming a Type‐II heterojunction; these factors facilitate the charge transfer and charge separation that induces photogenerated electrons to transfer from the CB of g‐C_3_N_4_ to the CB of TiO_2_.^[^ ^86^ ^]^ Recently, an investigation on ternary rGO@g‐C_3_N_4_/ZnO by Saeed et al.^[^ ^87^ ^]^ revealed increased photocatalytic MB degradation of 91.5% under visible light. The experimental finding showed an improved ability to harvest visible light absorption due to the addition of rGO and g‐C_3_N_4_ to ZnO by reducing the charge recombination. Representative works on S–(RGO/metal)–g‐(C_3_N_4_) are summarized in **Table** **5**.

**Table 5:** Summary of representative S–(RGO/metal)–g‐(C_3_N_4_) photocatalyst junction publications

Model/sample	Oxidation site	Reduction site	Mediator	Charge‐transfer direction across the junction	Activity test/application	References
g‐C_3_N_4_/CdS@rGO	CdS	g‐C_3_N_4_	RGO	CdS 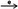 RGO, g‐C_3_N_4_ 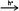 RGO	CO_2_ reduction	[334]
g‐C_3_N_4_/GO/AgBr	AgBr	g‐C_3_N_4_	GO	Ag 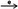 BrGO, g‐C_3_N_4_ 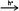 GO	Degradation of RhB	[335]
g‐C_3_N_4_/Au/CdS	CdS	g‐C_3_N_4_	Au	CdS 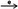 Au, g‐C_3_N_4_ 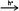 Au	Degradation of RhB, H_2_ production	[84, 336]
g‐C_3_N_4_/GO/TiO_2_	TiO_2_	g‐C_3_N_4_	GO	TiO_2_ 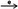 GO, g‐C_3_N_4_ 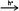 GO	Degradation of MB	[85]
g‐C_3_N_4_/Ag/BiVO_4_	BiVO_4_	g‐C_3_N_4_	Ag	BiVO_4_ 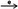 Ag, g‐C_3_N_4_ 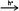 Ag	Decomposition of TC	[337]
g‐C_3_N_4_/RGO**/**Bi_2_WO_6_	Bi_2_WO_6_	g‐C_3_N_4_	RGO	Bi_2_WO_6_ 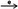 RGO, g‐C_3_N_4_ 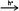 RGO	Degradation of 2,4,6‐trichlorophenol (TCP)	[338]
g‐C_3_N_4_/RGO /Bi_2_MoO_6_	Bi_2_MoO_6_	g‐C_3_N_4_	RGO	Bi_2_MoO_6_ 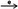 RGO, g‐C_3_N_4_ 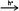 RGO	Degradation of RhB	[339]
g‐C_3_N_4_/RGO/CdS	CdS	g‐C_3_N_4_	RGO	CdS 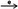 RGO, g‐C_3_N_4_ 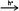 RGO	H_2_ generation and degradation of atrazine	[340]
g‐C_3_N_4_/BiOI/RGO	g‐C_3_N_4_	BiOI	RGO	g‐C_3_N_4_ 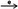 RGO, BiOI 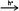 RGO	CO_2_ reduction	[341]
Co_3_O_4_–rGO–gC_3_N_4_	Co_3_O_4_	g‐C_3_N_4_	RGO	Co_3_O_4_ 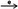 RGO, g‐C_3_N_4_ 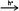 RGO	H_2_ generation	[342]
g‐C_3_N_4_/RGO/NiFe_2_O_4_	NiFe_2_O_4_	g‐C_3_N_4_	RGO	NiFe_2_O_4_ 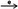 RGO, g‐C_3_N_4_ 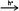 RGO	Degradation of MO	[343]
g‐C_3_N_4_/Cu/Cu_2_O	g‐C_3_N_4_	Cu_2_O	Cu	g‐C_3_N_4_ 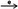 Cu, Cu_2_O 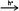 Cu	Degradation of MO and phenol	[344]
g‐C_3_N_4_/Ag/MoS_2_	MoS_2_	g‐C_3_N_4_	Ag	g‐C_3_N_4_ 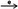 Ag, Cu_2_OAg, Ag 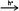 MoS_2_	Degradation of RhB	[345]
g‐C_3_N_4_/Au/CdZnS	CdZnS	g‐C_3_N_4_	Au	CdZnS 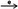 Au, g‐C_3_N_4_ 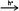 Au	H_2_ generation	[346]
g‐C_3_N_4_/Ag /Ag_3_VO_4_	Ag_3_VO_4_	g‐C_3_N_4_	Ag	Ag_3_VO_4_ 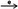 Au, g‐C_3_N_4_ 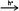 Au	Degradation of RhB	[347]
g‐C_3_N_4_–Ag–AgVO_3_	AgVO_3_	g‐C_3_N_4_	Ag	AgVO_3_ 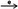 Ag, g‐C_3_N_4_ 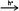 Ag	Degradation of RhB and bacterial inactivation	[348]
g‐C_3_N_4_/Ag/Ag_3_PO_4_	Ag_3_PO_4_	g‐C_3_N_4_	Ag	Ag_3_PO_4_ 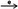 Ag, g‐C_3_N_4_ 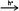 Ag	Degradation of RhB	[349]
g‐C_3_N_4_–Bi–BiOCl	BiOCl	g‐C_3_N_4_	Bi	BiOCl 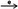 Bi, g‐C_3_N_4_ 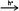 Bi	Degradation of RhB and Cr(VI) reduction	[350]
g‐C_3_N_4_/Ag/Ag_2_CO_3_	Ag_2_CO_3_	g‐C_3_N_4_	Ag	Ag_2_CO_3_ 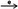 Ag, g‐C_3_N_4_ 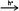 Ag	Degradation of RhB	[351]
g‐C_3_N_4_/Au/ZnIn_2_S_4_	ZnIn_2_S_4_	g‐C_3_N_4_	Au	ZnIn_2_S_4_ 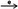 Au, g‐C_3_N_4_ 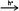 Au	Nitric oxide removal and carbon dioxide conversion	[352]
g‐C_3_N_4_/MoS_2_(Ni, Co)	MoS_2_	MoS_2_	–	g‐C_3_N_4_ 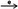 MoS_2_, g‐C_3_N_4_ 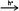 MoS_2_	H_2_ generation	[353]

## 9 Conclusions and Outlook

In this critical review, we have first presented an overview and introduction of photocatalysis using typical bare semiconductor and semiconductor‐based heterojunction photocatalysts, and then thoroughly reviewed current state‐of‐the‐art heterojunction photocatalytic devices including semiconductors with graphene, CQDs, and graphitic carbon nitride. The review's primary focus—on the steering of charge kinetics, construction principles, and mechanisms of heterojunction photocatalysis—is presented as an elaborative tutorial on the advantages and disadvantages of Schottky/ohmic and plasmonic junctions. Even though our review primarily focuses on theoretical modeling principles and recent progress in charge transfer and separation proposals for enhanced heterojunction photocatalysis, it also combines experimental observations in parallel for better understanding and inspiration.

Although photocatalysts hold promise for enabling a sustainable future world, there are several roadblocks preventing their use in practical applications. Notable factors that can hinder the achievement of highly efficient photocatalysis include: 1) failure to size the photocatalyst bandgap small enough so that the energies needed to convert photons into e–h pairs match solar spectrum irradiance; 2) an excessively high recombination rate; 3) insufficiently large surface area; 4) insufficiently high chemical stability; 5) suboptimum band positions of the photocatalyst; 6) insufficiently low cost.

To alleviate these shortcomings, a number of well‐known designs have been proposed as holding promise; apart from ones utilizing a single semiconductor—these designs either incorporate the interfacing of a semiconductor with metal, semiconductor–semiconductor, semiconductor–graphene, semiconductor–graphitic carbon nitride, or use metal and RGO as a mediator in the heterojunction structure. Controlling the formation and making use of the advantages of semiconductor–metal designs—with emphasis on their Schottky barrier/ohmic contacts and plasmonic effects—have been the main areas of focus in the past few years. Despite great effort, inadequate charge separation and loss of energy during migration of charge carriers from semiconductor to metal greatly quench the redox ability of these photocatalysts. Recently, a conventional Z‐scheme that uses acceptor and donor mediators and an all‐solid‐state Z‐scheme that uses metals as a mediator have been investigated, with no emphasis to date on the selective steering of charges. In general, Z‐scheme designs mainly aim to minimize both the recombination rate and lattice mismatch at the p–n junction and improve charge transfer dynamics. Recently, a few promising experimental and theoretical simulation studies have been undertaken to enhance the selective steering of charges in some all‐solid‐state Z‐scheme designs to increase the efficiency of charge separation for improved optoelectronic conversion.

Also recently, heterojunction photocatalysis studies have been extended to consider semiconductor–RGO–graphene, graphene–graphitic carbon nitride, and graphene–graphitic carbon nitride designs for better optimization of metal‐free, inexpensive, and environmentally harmless heterojunctions. Such heterojunctions mainly aim to maximize quantum efficiency by increasing the surface area of the photocatalyst, broadening the range of light absorption, and increasing the rate of charge transfer across the interface. Although few studies on the incorporation of CQDs with carbon nitride have been performed, the results reveal interesting effects and attain enhanced quantum efficiency, identifying this design as an emerging promising candidate for heterojunction photocatalysis.

Despite some success in heterojunction photocatalysis design and tuning charge kinetics dynamics, this area of development still faces many challenges. First, the interplay of dynamics at the interface is not sufficiently considered; this process can quench photocatalyst performance. Primarily, the connecting of the energy‐dependent selective steering of charges that facilitates charge migration to the ultimate goal of efficient charge separation has not received enough attention. Another challenge is the failure to apply experimental and theoretical principles at the interface to take proper advantage of the properties of Schottky/ohmic and plasmonic junctions. Especially, insufficient effort has been made at applying the merits of a metal's SPR effect so as to make use of its dual role. Even though experimental and theoretical study methods show advances in studying dynamical properties at surfaces and interfaces, they are limited. Most studies do not present refined charge kinetics dynamics at the interface, especially those related to charge transfer and high energy e–h pair separation; these areas need much more work in the future. In general, to further insight into the area of semiconductor based heterojunctions, the following points can be considered. 1) Less lattice mismatch at the interface with favorable band alignment between different combining layers is crucial. In addition, the area should also focus on an energy‐dependent Z‐scheme p–m–n and n–m–p heterojunction to steer charges selectively across the interface to achieve good charge separation; and 2) In synthesizing metal‐free photocatalysts, combining g‐C_3_N_4_ with large bandgap semiconductors is a promising strategy to extend its light absorption region and increase its surface area. In addition, as experts and reviewers of this area, we grasp that the proportional composition of materials at the interface, their morphology, and crystal structures have not been properly considered, despite the fact that these properties greatly affect the photocatalytic activity of the photocatalyst.

## Conflict of Interest

The authors declare no conflict of interest.
